# 3D mesoporous graphene nanoparticle-mediated transient silencing of susceptibility genes confers broad-spectrum disease resistance in crops

**DOI:** 10.1016/j.xplc.2026.101983

**Published:** 2026-06-29

**Authors:** Wangshan Lu, Yule Ye, Chao Wang, Yizhou Hao, Zhaojie Chen, Zhuoyi Lin, Jinbiao Ma, Yueyi Ren, Xinyu Liu, Dongong Niu, Zhengguang Zhang, Jianhao Chen, Gan Ai, Daolong Dou

**Affiliations:** 1 State Key Laboratory of Agricultural and Forestry Biosecurity, College of Plant Protection, Academy for Advanced Interdisciplinary Studies, Nanjing Agricultural University, Nanjing, China; 2 Guangzhou Moxi Technology Co Ltd., Guangzhou 510535, China; 3 Guangxi Key Laboratory of Agric-Environment and Agric-Products Safety, College of Agriculture, Guangxi University, Nanning 530004, Guangxi, China; 4 State Key Laboratory of Wheat Improvement, College of Life Sciences, Shandong Agricultural University, Tai’an 271018, China; 5 International Center for Quantum Materials, School of Physics, Peking University, Beijing 100871, China

**Keywords:** RNAi, mesoporous graphene, susceptibility genes, broad-spectrum resistance

## Abstract

Gene editing of plant susceptibility (S) genes can enhance crop disease resistance, but permanent disruption of these genes often causes undesirable side effects, including reduced growth and yield. Here, we developed three-dimensional mesoporous graphene nanoparticles (MGNs) for the protection and delivery of double-stranded RNA (dsRNA) into plant cells, enabling transient silencing of S genes. MGNs showed high colloidal stability, strong dsRNA-binding affinity, and effective protection of dsRNA against UV exposure, RNase degradation, and heat. In *Nicotiana benthamiana*, MGN–dsRNA treatment achieved up to 78% target-gene silencing and significantly enhanced resistance to *Phytophthora capsici* without growth penalties when applied at optimized intervals. In rice, transient silencing of the S gene *ROD1* using MGN–dsRNA conferred broad-spectrum resistance to both *Magnaporthe oryzae* and *Xanthomonas oryzae* pv. *oryzae* in growth-chamber and field trials, while also increasing yield relative to controls. This nanoparticle-enabled transient gene-silencing strategy provides a rapid, non-transgenic approach for improving crop disease resistance and may help avoid the trade-offs associated with permanent S-gene modification.

## Teaser

Permanent modification of plant susceptibility (S) genes can enhance disease resistance but often comes at the expense of plant growth and yield. This study presents a three-dimensional mesoporous graphene nanoparticle platform that protects and delivers double-stranded RNA into plant cells, enabling efficient and transient S-gene silencing. This non-transgenic approach confers broad-spectrum disease resistance in both model plants and field-grown crops while potentially avoiding the trade-offs associated with permanent S-gene modification.

## Introduction

Plant diseases reduce global crop production by 20% to 30% each year, posing a major threat to food security worldwide (Savary et al., 2019; Jones et al., 2024). One emerging strategy for improving crop disease resistance is the genetic disruption of susceptibility (S) genes, which encode host factors that pathogens exploit to establish infection (Zaidi et al., 2018; Li et al., 2021b; Garcia-Ruiz et al., 2021). However, three major limitations have restricted the use of this strategy in commercial agriculture. First, permanent disruption of S genes can cause unintended side effects, including constitutive immune activation and impaired development or yield (Zaidi et al., 2018; Garcia-Ruiz et al., 2021; Li et al., 2021b). Second, introducing suitable S-gene edits into different cultivars requires considerable time and resources. Third, genetically modified and gene-edited crops remain subject to strict regulatory restrictions in many countries. Together, these limitations highlight the need for alternative strategies that can target S genes without permanently modifying the plant genome.

Spray-induced gene silencing (SIGS) is a promising approach for disease control that uses double-stranded RNAs (dsRNAs) or small RNAs (sRNAs) to silence essential pathogen genes (Wang and Jin, 2017; Qiao et al., 2021). Compared with conventional fungicides, SIGS offers several environmental advantages, including high target specificity and limited environmental persistence (Wang and Jin, 2017; Qiao et al., 2021). However, this same target specificity can limit its ability to provide broad-spectrum resistance with a single dsRNA design. We therefore hypothesized that SIGS could be redirected from pathogen targets to plant S genes, enabling transient host-gene silencing and broad-spectrum disease resistance while avoiding the drawbacks associated with permanent genetic modifications.

Testing this hypothesis required an effective dsRNA-delivery system. When dsRNA is applied to crops, nanocarriers can promote cellular uptake and protect RNA molecules from environmental degradation, particularly UV-induced photodegradation (Mosquera et al., 2025). Several nanocarriers, including clay nanosheets (Mitter et al., 2017b), DNA nanostructures (Zhang et al., 2019), and carbon dots (Schwartz et al., 2020), have been developed for RNA protection and delivery in plants. Graphene-based nanocarriers are also promising vectors for plant gene silencing (Verma et al., 2019). Owing to their zero-band-gap electronic structure, graphene materials exhibit strong UV absorption, making them attractive candidates for UV-shielding additives and protective coatings (Murariu et al., 2021; Kuo et al., 2022). Carbon nanotubes and graphene oxide nanoparticles have been used for sRNA delivery and localized silencing of plant genes (Demirer et al., 2020; Li et al., 2022b). However, some carbon-based nanomaterials may cause cytotoxicity in organisms, in part because of their high aspect ratios or sharp edges (Radomski et al., 2005; Poland et al., 2008; Tu et al., 2013). This potential limitation underscores the need for biocompatible nanocarriers that combine efficient RNA loading, environmental protection, and safe delivery into plant cells.

In this study, we developed three-dimensional (3D) mesoporous graphene nanoparticles (MGNs) as a biocompatible and highly stable nanocarrier system for dsRNA protection and delivery in plants. We show that MGNs efficiently bind dsRNA, protect it from enzymatic and environmental degradation, and promote its uptake in both model and crop plants. By delivering dsRNA that transiently silences plant S genes, this approach enhances broad-spectrum disease resistance in *Nicotiana benthamiana* and rice under laboratory, growth-chamber, and field conditions. Importantly, transient S-gene silencing improved disease resistance without the growth penalties associated with permanent gene disruption. Our findings establish MGN-mediated dsRNA delivery as a versatile and rapidly deployable strategy for improving crop resilience through nanoparticle-enabled transient gene silencing.

## Results

### Synthesis and physicochemical characterization of MGNs

**Figure 1:**
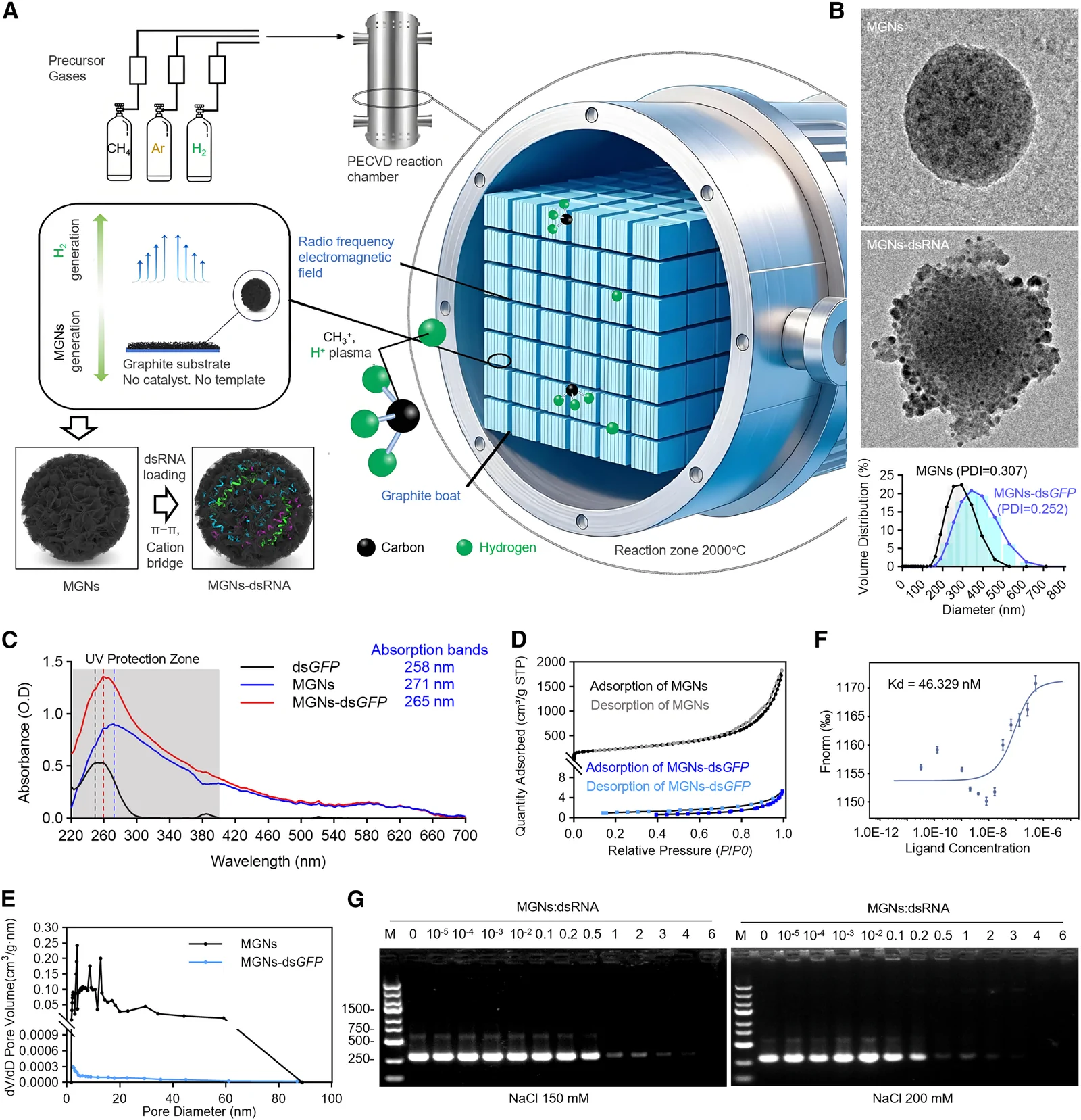
Synthesis and physicochemical characterization of MGNs. **(A)** Schematic illustration of the catalyst-free PECVD method used to synthesize MGNs. **(B)** TEM images and hydrodynamic diameter distributions of MGNs and MGN−dsGFP complexes. Hydrodynamic diameter distributions are shown below the corresponding TEM images. **(C)** UV-visible absorption spectra of naked dsGFP, MGNs, and MGN–dsRNA complexes. **(D)** Nitrogen adsorption–desorption isotherms of MGNs and MGN–dsGFP complexes. The horizontal axis shows the relative pressure (*P*/*P*_0_), and the vertical axis shows the quantity adsorbed (cm^3^/g STP). **(E)** Pore-size distributions of MGNs and MGN–dsGFP complexes. The horizontal axis shows pore diameter (nm), and the vertical axis shows differential pore volume (d*V*/d*D*, cm^3^/g·nm). **(F)** Microscale thermophoresis analysis of the binding affinity between MGNs and ds*GFP*. The saturation-binding curve was fitted by non-linear regression, yielding a dissociation constant (*K*_D_) of 46.33 nM. **(G)** Gel retardation assay showing the binding of MGNs to dsRNA at different MGN-to-dsRNA mass ratios in the presence of 150 or 200 mM NaCl.

The 3D MGNs were synthesized using a catalyst-free plasma-enhanced chemical vapor deposition (PECVD) approach (Figure 1A and supplemental information). This approach enables the production of high-purity MGNs without pollutant emissions, while simultaneously generating hydrogen as a clean-energy by-product (Figure 1A). Dynamic light scattering (DLS) analysis showed that the synthesized MGNs had an average hydrodynamic diameter of approximately 280 nm, with sizes ranging from 140 to 540 nm (Figure 1B and Supplemental Figure 1A). This size range is above that of sub-100-nm nanoparticles, which have been associated with increased pulmonary and capillary accumulation risks (Chithrani et al., 2006; Nel et al., 2006; Oberdoester, 2010). The MGNs maintained a consistent hydrodynamic size after 1 month of storage in aqueous solution at room temperature, indicating high colloidal stability (Supplemental Figure 1B). UV-visible absorption spectroscopy confirmed the strong UV-shielding capacity of the MGNs (Figure 1C). Naked dsGFP (dsRNA targeting the green fluorescent protein gene used here as a model substrate) showed a characteristic absorption peak at approximately 260 nm (absorbance of approximately 0.53 a.u.). By contrast, pure MGNs displayed a broad and intense absorption band across the UV spectrum, with a peak absorbance of approximately 0.90 a.u. at 271 nm. This strong broadband optical attenuation suggests that MGNs could act as a physical barrier, protecting loaded dsRNA from UV-induced photochemical degradation.

To evaluate the structural suitability of MGNs for loading large biomolecules such as dsRNA, we characterized their pore structure using N_2_ adsorption and desorption isotherms. The MGNs exhibited a characteristic type IV isotherm with a distinct H3-type hysteresis loop, confirming a well-defined mesoporous architecture (Figure 1D). Their specific surface area, determined using the Brunauer-Emmett-Teller (BET) method, was 827.33 m^2^/g. Pore-size distribution (PSD) analysis using the Barrett–Joyner–Halenda (BJH) method revealed a multimodal distribution, with primary mesopore peaks at approximately 3.9 nm and 12.8 nm, together with a population of larger mesopores (> 50 nm) (Figure 1E). This hierarchical porous structure provides a large accessible surface area that is well suited for high-capacity loading of macromolecular dsRNA.

### Efficient encapsulation of dsRNA within MGNs

Graphene-based materials exhibit a strong propensity for π–π interactions (Varghese et al., 2009; Georgakilas et al., 2012). Because transient breathing of the dsRNA double helix can expose internal nucleobases (Snoussi and Leroy, 2001), these bases may interact with the graphitic surface of MGNs through π–π stacking. We quantified this interaction using microscale thermophoresis (MST) with dsGFP, which revealed a strong binding affinity between MGNs and dsGFP, with a dissociation constant (*K*_D_) of 46.3 nM (Figure 1F). Consistent with this result, gel retardation assays showed that an MGN-to-dsRNA mass ratio of only 1:1 was sufficient to achieve near-complete dsRNA loading (Figure 1G).

Both dsRNA and MGNs carry a strong negative surface charge (MGN zeta potential = −37.94 mV; Supplemental Figure 1C), creating an electrostatic barrier to complex formation. Because the dsRNA was prepared in Tris-buffered saline (TBS) containing 150 mM Na^+^, we hypothesized that sodium ions facilitate complex formation by screening the surface charges on both components, thereby reducing electrostatic repulsion and allowing π–π interactions to predominate (Wu et al., 2011). Consistent with this hypothesis, increasing the NaCl concentration from 150 mM to 200 mM significantly improved dsRNA loading efficiency (Figure 1G), supporting a role for Na^+^ in promoting MGN−dsRNA complex formation.

The formation of MGN–dsRNA complexes was further confirmed using complementary physicochemical analyses. Transmission electron microscopy (TEM) showed that dsRNA associated with the MGN surface, and DLS revealed the expected increase in average hydrodynamic diameter from approximately 280 nm to 353 nm following dsRNA loading (Figure 1B). UV-visible spectroscopy showed a 7 nm bathochromic (red) shift in the maximum absorption wavelength relative to free dsRNA, indicating changes in the electronic environment upon complex formation (Figure 1C). In addition, BET and PSD analyses revealed marked reductions in both specific surface area and pore volume after dsRNA loading, consistent with occupation of the mesoporous structure by dsRNA (Figures 1D and 1E). The zeta potential changed only slightly after complex formation, from −37.94 mV to −38.66 mV (Supplemental Figure 1C), indicating that the nanoparticles retained a highly negative surface charge (< −30 mV) associated with good colloidal stability (Kaur and Tikoo, 2013; Duval et al., 2019). Consistent with this observation, MGN–dsGFP complexes maintained an average hydrodynamic diameter of approximately 339 nm after 3 days of storage (Supplemental Figure 1D).

We next examined the *in vitro* release kinetics of dsRNA from MGN–dsRNA complexes. At pH 7.5 (representing the storage buffer), dsRNA release was notably slow, with only approximately 34% of the cargo released over 120 h (Supplemental Figure 1E). By contrast, at pH 5.5, which approximates the acidic environment of the plant apoplast (Geilfus, 2017), cumulative dsRNA release reached approximately 53.6% within 72 h (Supplemental Figure 1E). These results indicate that the complexes remain relatively stable during storage while promoting greater dsRNA release under acidic conditions. Together, these findings demonstrate that MGNs efficiently encapsulate dsRNA and provide a stable nanocarrier for its delivery.

### MGNs protect dsRNA from environmental degradation

**Figure 2:**
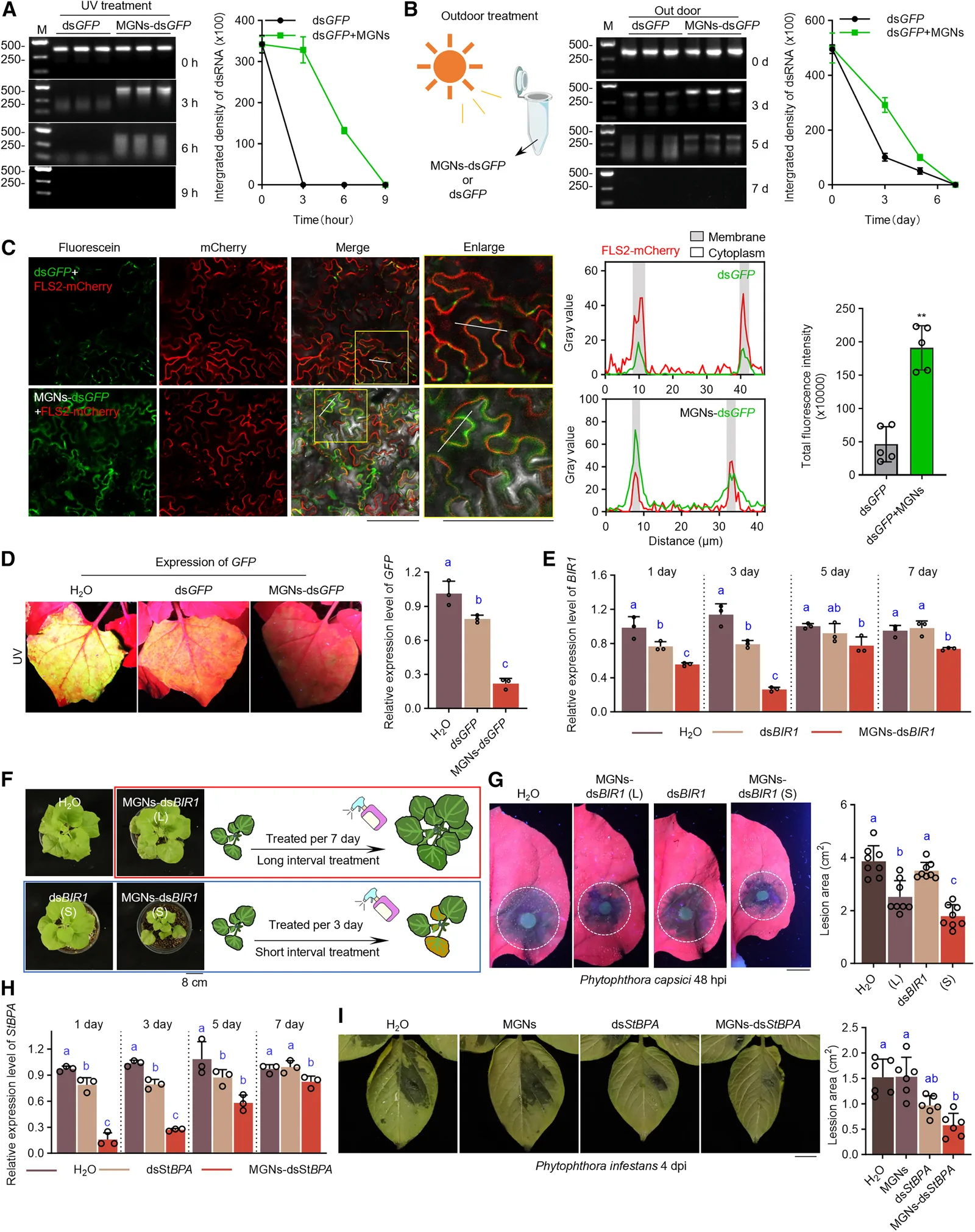
MGN–dsRNA-mediated S-gene silencing enhances disease resistance while limiting growth penalties. **(A)** Protection of dsRNA from UV-induced degradation by MGNs. The integrity of naked dsGFP and MGN–dsGFP complexes, each containing a final dsGFP concentration of 50 ng/μl, was assessed after 3, 6, or 9 h of UV irradiation. Band intensities were quantified by measuring total fluorescence intensity. Data are means ± SD (n = 3 biological replicates). **(B)** Protection of dsRNA by MGN under outdoor conditions. A schematic of the outdoor exposure experiment is shown on the left. Data are means ± SD (*n* = 3). **(C)** MGN-mediated delivery of dsRNA into *N. benthamiana leaf cells*. The line-scan profile in the middle shows the fluorescence intensities of FLS2 and dsGFP along the transect indicated in the images on the left. Data are means ± SD (*n* = 3; Student’s *t-*test, ∗∗*p* < 0.01). Scale bar, 100 μm. **(D)** GFP fluorescence in GFP-overexpressing *N*. *benthamiana* leaves following foliar application of H_2_O, naked dsGFP, or MGN–dsGFP complexes. Relative GFP transcript levels determined by RT–qPCR are shown on the right. Data are means ± SD (*n* = 3; one-way analysis of variance (ANOVA), *p* < 0.05). **(E)** Relative NbBIR1 transcript levels in *N*. *benthamiana* leaves after foliar application of H_2_O, naked dsBIR1, or MGN–dsBIR1 complexes. Data are means ± SD (*n* = 3; one-way ANOVA, *p* < 0.05). **(F)** Growth phenotypes of NbBIR1-silenced plants subjected to long- or short- interval applications. The application schedules are illustrated on the right. **(G)** Disease symptoms in *N*. *benthamiana* leaves inoculated with *Phytophthora capsici* after long- or short-interval application of MGN–dsBIR1*.* Plants were inoculated 24 h after the final application. Data are means ± SD (*n* = 8; one-way ANOVA, *p* < 0.05). Scale bar, 1 cm. **(H and I)** MGN–dsRNA–mediated silencing of *StBPA* enhances potato resistance to *P*. *infestans*. Relative StBPA transcript levels in potato leaves after foliar application of MGN–dsStBPA complexes are shown in **(H)**. Disease symptoms and lesion areas in plants inoculated with *P. infestans* 1 day after foliar application of water, MGNs, naked dsStBPA, or MGN–dsStBPA complexes **(I)**. Data are means ± SD (*n* = 6; one-way ANOVA, *p* < 0.05). Scale bar, 1 cm.

Field application of dsRNA requires protection against UV radiation, RNA-degrading enzymes, and high temperatures encountered under natural conditions (Sang and Kim, 2020). We first examined whether MGNs protected dsRNA from UV-induced degradation, using naked dsRNA as a control. Gel electrophoresis showed that MGN-loaded dsRNA remained largely intact after 3 h of UV exposure (Figure 2A). MGNs also protected dsRNA from degradation by RNase (Supplemental Figure 2A), plant extracts (Supplemental Figure 2B), and heat treatment (Supplemental Figure 2C). To assess protection under more realistic environmental conditions, we exposed MGN–dsGFP complexes to outdoor conditions (Figure 2B). MGN-loaded dsRNA remained detectable for up to 5 days and showed substantial degradation by day 7, whereas naked dsRNA was degraded within 3 days. These results indicate that MGNs improve the environmental stability of dsRNA.

We next compared the protective capacity of MGNs with that of three established nanocarriers: carbon quantum dots (CQDs) (Schwartz et al., 2020; Jiang et al., 2026), polyethylenimine (PEI) (Wang et al., 2023b), and mesoporous silica nanoparticles (MSNs) (Cai et al., 2024). Under prolonged UV exposure, MGNs preserved dsRNA integrity more effectively than the other tested materials, although MSNs also provided substantial protection (Supplemental Figure 2D). A similar pattern was observed after heat treatment at 95°C, with MGNs showing comparatively greater preservation of dsRNA integrity (Supplemental Figure 2E). Because nanocarrier performance can vary with synthesis conditions and production batches, these comparisons reflect the materials tested under the specific conditions used in this study.

### MGNs enhance dsRNA delivery and gene-silencing efficiency

We next examined the delivery efficiency of MGN–dsRNA complexes in the model plant *N. benthamiana* by using fluorescently labeled dsGFP. Compared with naked dsGFP, MGN-loaded dsGFP showed approximately 6.43-fold and 4.46-fold higher fluorescence intensities in *N*. *benthamiana* protoplasts and epidermal cells, respectively (Supplemental Figure 3A and Figure 2C). MGNs also increased dsGFP accumulation in mesophyll cells by approximately 3.57-fold relative to naked dsGFP (Supplemental Figure 3B), indicating that MGN-mediated delivery extended beyond the leaf epidermis. To distinguish intracellular uptake from surface adsorption, we used FLS2-mCherry as a plasma membrane marker (Li et al., 2022d). Line-scan analysis showed that the fluorescence profile of MGN-loaded dsGFP extended beyond that of the FLS2-mCherry signal, consistent with dsRNA entry across the plasma membrane and into the cell interior (Figure 2C). Quantitative colocalization analysis showed a correlation (*r* = 0.653) between the MGN-loaded dsGFP and FLS2-mCherry signals (Supplemental Figure 3C), supporting close association with the plasma membrane and partial intracellular localization. The delivery efficiency of MGNs was comparable to that of CQDs, MSNs, and PEI under the tested conditions (Supplemental Figure 3D).

To evaluate gene-silencing efficiency, we sprayed MGN–dsGFP onto leaves expressing GFP. Naked dsGFP reduced GFP transcript abundance by approximately 21%, whereas MGN–dsGFP reduced it by approximately 78% (Figure 2D). Consistent with these results, leaves treated with MGN–dsGFP showed markedly lower GFP fluorescence than control leaves and leaves treated with naked dsGFP (Figure 2D and Supplemental Figure 3E). Western blot analysis further showed reduced GFP protein accumulation following MGN–dsGFP treatment, confirming the high silencing efficiency of the MGN–dsRNA delivery system (Supplemental Figure 3F).

### MGN–dsRNA targeting S genes enhances plant disease resistance

Disruption of S genes can cause constitutive immune activation and impaired plant growth (Gao et al., 2021; Wu et al., 2024). Because MGN–dsRNA-mediated silencing is transient, we reasoned that optimizing the interval between applications might enhance disease resistance without compromising growth. To test this possibility, we designed dsRNA targeting *BAK1-interacting receptor-like kinase 1* (*BIR1*), which encodes a key negative regulator of plant immunity. Mutation or virus-induced silencing of *BIR1* causes constitutive immune activation and a dwarf phenotype (Supplemental Figure 4A) (Gao et al., 2009). RT–qPCR analysis showed that MGN–dsBIR1 treatment reduced *BIR1* transcript abundance to approximately 20% of the control level by day 3, after which expression recovered to approximately 70% by day 5, confirming that the silencing effect was transient (Figure 2E). We also compared *BIR1* silencing mediated by different nanocarriers. MGNs, CQDs, and MSNs each reduced BIR1 transcript abundance by more than 70% at day 3, followed by a similar recovery by day 5 (Supplemental Figure 4B), indicating that transient S-gene silencing can be achieved using several nanocarrier systems. Because reactive oxygen species (ROS) contribute to the activation of plant immune responses (Wu et al., 2023), we next examined ROS accumulation following *NbBIR1 silencing. 3,3′-Diaminobenzidine (*DAB) staining showed that MGN–dsBIR1 induced ROS accumulation during the first 3 days, which subsequently declined by day 5 (Supplemental Figure 4C). We therefore applied MGN–dsBIR1 every 7 days as a long-interval treatment or every 3 days as a short-interval treatment designed to maintain continuous silencing (Figure 2F). Short-interval application of MGN–dsBIR1 caused pronounced dwarfism and sustained ROS accumulation, whereas long-interval application resulted in transient ROS accumulation without detectable adverse effects on plant growth (Figure 2F and Supplemental Figure 4C). Importantly, long-interval treatment still significantly increased resistance to *Phytophthora capsici* (Figure 2G). These results show that optimizing the application interval enables transient S-gene silencing to enhance disease resistance while minimizing growth penalties.

To evaluate the effectiveness of MGN-mediated S-gene silencing in a crop species, we applied MGN–dsRNA targeting *StBPA*, a known susceptibility gene in *Solanum tuberosum* (potato) (Li et al., 2025). Time-course expression analysis showed that MGN–dsStBPA reduced StBPA transcript abundance by more than 70%, with strong silencing maintained for up to 3 days (Figure 2H). Consistent with this reduction, disease assessment at 4 days post-inoculation (dpi) with *Phytophthora infestans* showed enhanced resistance to late blight (Figure 2I). Leaves treated with MGN–dsStBPA developed more restricted symptoms and significantly smaller lesions than those treated with water, empty MGNs, or naked ds*StBPA* (Figure 2I). Together, these results demonstrate that MGN-mediated dsRNA delivery can transiently silence host susceptibility genes and enhance resistance to plant pathogens.

### Field application of MGN–dsRNA promotes rice resistance to *Magnaporthe oryzae* and *Xanthomonas oryzae* pv. *oryzae*

**Figure 3:**
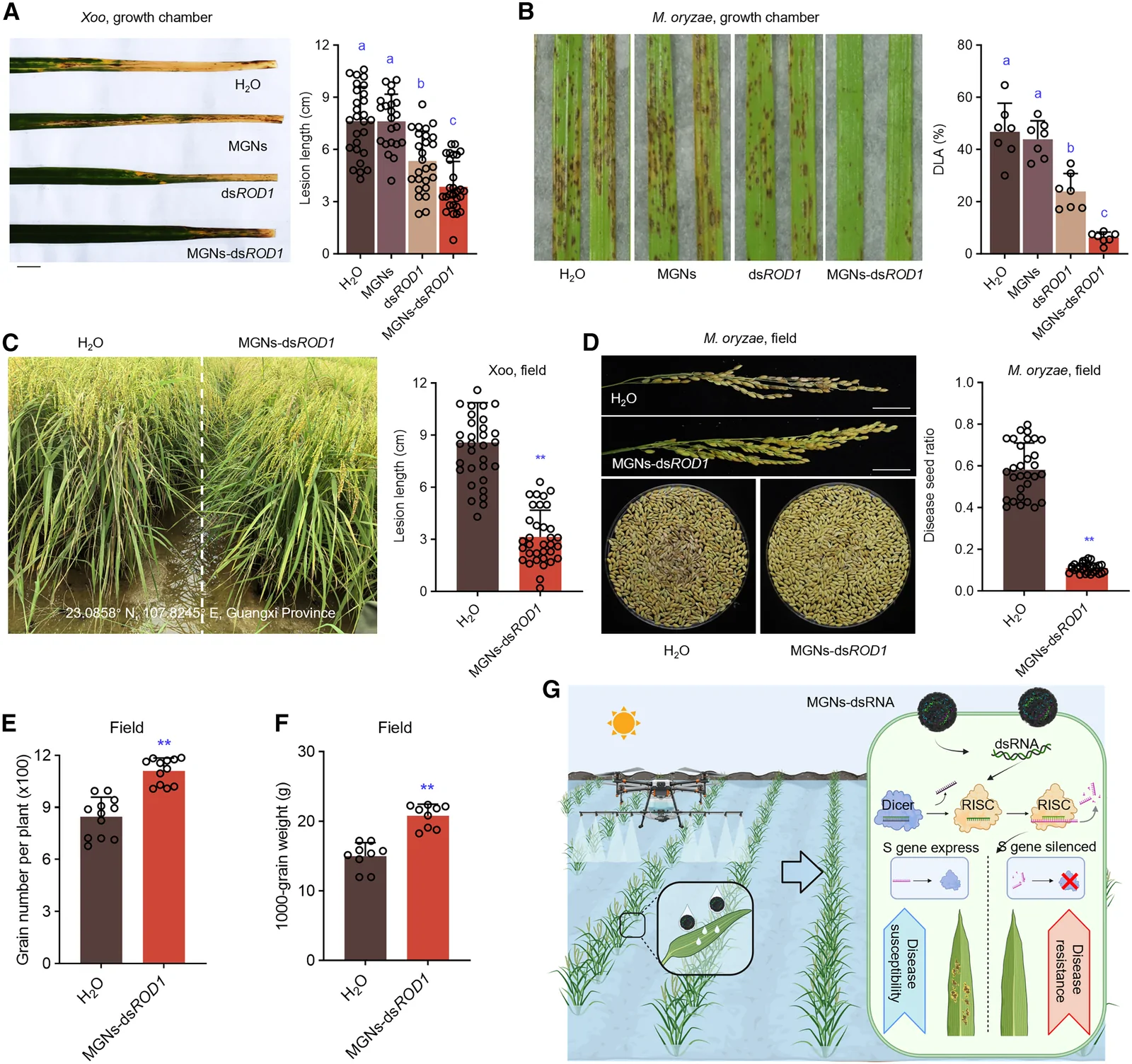
MGN–dsROD1-mediated *ROD1* silencing enhances resistance to multiple pathogens under field conditions. **(A)** Disease symptoms of rice following infection with *Xoo*. Lesion lengths are shown on the right. Data are means ± SD (*n* > 21 biological replicates; one-way ANOVA, *p* < 0.05). Scale bar, 1 cm. **(B)** Disease symptoms of rice following infection with *M*. *oryzae*. Disease lesion area (%) is shown on the right. Data are mean ± SD (*n* > 21 biological replicates; one-way ANOVA, *p* < 0.05). **(C and D)** Field evaluation of MGN–dsROD1-mediated disease resistance. *Xoo* lesion lengths are shown in **(C)** (means ± SD, *n* = 34; Student’s *t-*test, ∗∗*p* < 0.01). Disease incidence caused by *M*. *oryzae* is shown in **(D)** (means ± SD; *n* = 30; Student’s *t-*test, ∗∗*p* < 0.01). Scale bar, 3 cm. **(E and F)** Effects of MGN–dsROD1 treatment on rice yield. Grain number per plant **(E)** and 1,000-grain weight **(F)** are shown. Data are means ± SD (one-way ANOVA, *p* < 0.05). **(G)** Proposed model illustrating enhanced broad-spectrum disease resistance following MGN-mediated delivery of dsRNA targeting plant S genes.

We next evaluated the practical application of MGN–dsRNA in rice, in which *ROD1* functions as an S gene. Deletion of *ROD1* enhances resistance to multiple diseases, including rice blast caused by the fungal pathogen *Magnaporthe oryzae* and bacterial blight caused by *Xanthomonas oryzae* pv. *oryzae* (*Xoo*), but also results in significant yield penalties (Gao et al., 2021; Wu et al., 2024). RNA-delivery experiments showed that MGNs efficiently delivered dsROD1 into rice cells (Supplemental Figure 5A). MGN–dsROD1 treatment maintained more than 50% *ROD1* silencing for 1 week after application (Supplemental Figure 5B). Among the nanocarriers tested, MGNs and CQDs showed greater silencing efficiency and durability than PEI- and MSN-based carriers (Supplemental Figure 5C). A single application of MGN–dsROD1 induced transient ROS accumulation, with ROS levels peaking at day 3 and returning to basal levels by days 5 to 7, and also increased the expression of the defense marker gene *OsPR1a*, indicating activation of plant immune responses (Supplemental Figures 5D and 5E). Consistent with these responses, MGN–dsROD1 enhanced rice resistance to both *M*. *oryzae* and *Xoo* under growth-chamber conditions (Figures 3A and 3B).

To evaluate the effectiveness of this approach under field conditions, we conducted 2-year field trials in Guangxi (in 2025) and Hainan (in 2026) provinces, China. MGN–dsROD1 was applied every 10 days to maintain disease protection while minimizing potential effects on plant growth. In Guangxi, where both *Xoo* and *M*. *oryzae* were naturally present, MGN–dsROD1 treatment significantly enhanced disease resistance, reducing *Xoo* lesion length by approximately 64% and *M. oryzae* disease incidence by approximately 81% (Figures 3C and 3D). MGN–dsROD1 treatment also significantly increased grain yield compared with the water-treated control (Figures 3E and 3F), supporting the potential of transient S-gene silencing to improve resistance against multiple pathogens under field conditions. Similarly, in Hainan, where only *M*. *oryzae* infection occurred, MGN–dsROD1 reduced disease incidence to 55% and significantly increased rice yield (Supplemental Figures 6A–6E), further demonstrating the effectiveness of this transient silencing strategy under natural disease pressure.

### Biosafety evaluation of MGNs in plants, the phyllosphere microbiome, and animal models

**Figure 4:**
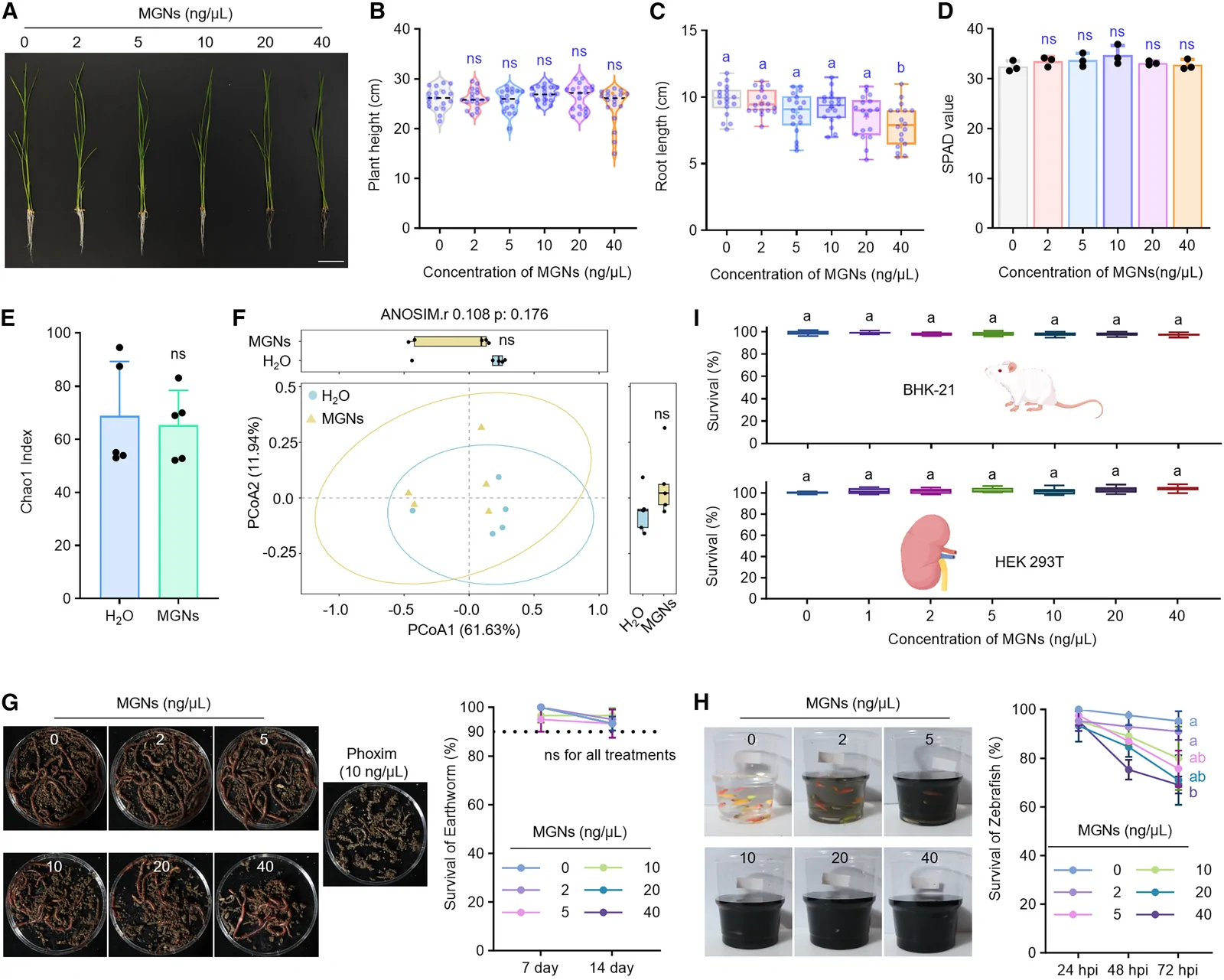
Biosafety evaluation of MGNs. **(A–D)** Growth responses of rice seedlings treated with different concentrations of MGNs. Germinated rice seeds were precultured in standard nutrient solution for 7 days and then transferred to nutrient solution containing MGNs at final concentrations of 0, 2, 5, 10, 20, or 40 ng/μl for an additional 7 days. Plant height **(B)**, primary root length **(C)**, and chlorophyll content **(D)** were measured after treatment. A slight reduction in root length was observed only at the higher MGN concentration of 40 ng/μl. Data are means ± SD (plant height and root length, *n* = 18; chlorophyll content, *n* = 3; one-way ANOVA, *p* < 0.05). Scale bar, 5 cm. **(E)** Alpha diversity of the phyllosphere bacterial community measured using the Chao1 index. Data are means ± SD (*n* = 5; Student’s *t-*test, ∗∗*p* < 0.01). **(F)** Principal-coordinate analysis (PCoA) of phyllosphere bacterial communities following H_2_O or MGN treatment. ANOSIM detected no significant difference in community structure between treatments (*R* = 0.108, *p* = 0.176; *n* = 5). **(G)** Environmental biosafety assessment of MGNs using earthworms. Representative images of earthworms exposed for 14 days to soils containing the indicated concentrations of MGNs (0–40 ng/μl) or 10 ng/μl Phoxim (positive control) are shown on the left. Survival rates at 7 and 14 days are shown on the right. The dotted line indicates a 90% survival threshold. Data are means ± SD (*n* = 3 independent biological replicates, with 20 earthworms per treatment per replicate; one-way ANOVA, *p* < 0.05). **(H)** Acute toxicity assessment of MGNs in zebrafish. Representative images are shown on the left, and survival rates over 72 h are shown on the right. Data are means ± SD from three independent experiments (*n* = 15 zebrafish per treatment per replicate; one-way ANOVA, *p* < 0.05). **(I)***In vitro* cytotoxicity assessment of MGNs in mammalian cells. Cell viability of BHK-21 and HEK293T cells was measured after 72 h of exposure to the indicated concentrations of MGNs. Data are means ± SD (*n* = 6; one-way ANOVA, *p* < 0.05).

We next evaluated the biosafety of MGNs in plants, associated microbial communities, and animal models. To assess phytotoxicity, rice seedlings were grown in medium containing increasing concentrations of MGNs (0–40 ng/μl), with 10 ng/μl corresponding to the concentration used in the field trials (Figure 4A). MGNs did not affect shoot height at any concentration tested (Figure 4B), whereas only the highest concentration (40 ng/μl) caused a modest reduction (approximately 20%) in root length (Figure 4C). SPAD measurements further showed that MGNs did not affect chlorophyll accumulation (Figure 4D). We also compared MGNs with PEI, CQDs, and MSNs to evaluate their effects on photosynthesis and stress responses. Measurements taken 3 days after treatment showed that MGNs did not reduce the net photosynthetic rate, whereas MSNs caused a slight decrease. In contrast, CQD and PEI treatments resulted in a modest increase in photosynthetic rate (Supplemental Figure 7A). Expression analysis of the defense marker *OsPR1a* and the stress-responsive genes *OsGLR28* and *OsWRKY71* further supported the biocompatibility of MGNs. MGN treatment did not significantly alter the expression of any of these genes, whereas MSNs moderately induced *OsWRKY71* expression and PEI upregulated all three genes (Supplemental Figures 7B–7D), consistent with the reported cytotoxicity of PEI (Fischer et al., 2003).

To assess potential ecological effects, we analyzed the leaf-associated bacterial community. Alpha diversity, measured using the Chao1 and ACE richness indices (Figure 4E and Supplemental Figure 7E) and the Shannon diversity index (Supplemental Figure 7F), did not differ significantly between water-treated and MGN-treated plants. Principal-coordinate analysis (PCoA) likewise showed substantial overlap between the two groups, with no clear separation along the principal axes (Figure 4F). This observation was supported by analysis of similarities (ANOSIM), which detected no significant difference in beta diversity (*r* = 0.108, *p* = 0.176). Unsupervised hierarchical clustering also showed extensive intermixing of water-treated and MGN-treated samples (Supplemental Figure 7G). Together, these results indicate that foliar application of MGNs had no detectable effect on overall composition or diversity of the phyllosphere bacterial community.

Finally, we evaluated the acute toxicity of MGNs in animal and mammalian models. For terrestrial ecotoxicity testing, earthworms were exposed to soils containing different concentrations of MGNs. No adverse effects on earthworm survival were observed, whereas treatment with the pesticide Phoxim (10 ng/μl) resulted in 100% mortality under the same conditions (Figure 4G). Similarly, acute toxicity assays in zebrafish detected no significant mortality at the concentration used in the field, with only the highest concentration causing a modest reduction (approximately 26%) in survival (Figure 4H). *In vitro* cytotoxicity assays using BHK-21 (mouse kidney) and HEK293T (human embryonic kidney) cells further showed that MGNs exhibited no detectable cytotoxicity at concentrations up to 40 ng/μl (Figure 4I). Together, these findings indicate that MGNs exhibit low toxicity under the tested conditions. Combined with their high efficiency for transient S-gene silencing, these properties make MGNs a promising nanocarrier platform for sustainable crop protection.

## Discussion

Genetic disruption of S genes is a promising strategy for enhancing crop disease resistance (Zaidi et al., 2018; Garcia-Ruiz et al., 2021; Li et al., 2021b). For example, loss-of-function mutations in *Mildew resistance locus O* (*MLO*) and *ROD1* confer broad-spectrum resistance to powdery mildew in wheat and to *Xoo and M. oryzae in* rice, respectively (Wang et al., 2014; Gao et al., 2021). However, permanent disruption of S genes often imposes fitness costs (e.g., in *mlo* and *rod1* mutants). Precision genome editing and the identification of favorable S-gene alleles can reduce these penalties (Gao et al., 2021; Li et al., 2022c); however, transferring such traits into diverse elite cultivars remains time-consuming. Regulatory restrictions on genetically modified and gene-edited crops may further limit their agricultural deployment. In this study, we used 3D MGNs to deliver dsRNA targeting S genes, thereby enabling transient and inducible gene silencing (Figures 1A and 3G). By optimizing the interval between applications, we enhanced disease resistance while minimizing growth inhibition (Figure 2F). This approach provides a flexible, non-transgenic alternative for exploiting S genes and could potentially be applied across different crop cultivars without the need for stable genetic modification. Several S genes, including *ACD11* and *BIR1, confer strong disease resistance when disrupted but also cause severe growth defects* (Brodersen et al., 2002; Gao et al., 2009). Transient silencing may therefore provide a practical means of exploiting such genes while limiting the developmental costs associated with their permanent loss.

The agricultural feasibility of nanocarrier-mediated dsRNA delivery depends not only on delivery efficiency but also on scalable, cost-effective, and environmentally compatible production (Chen et al., 2023). In this study, we synthesized MGNs using a PECVD approach that avoids harsh chemicals and noble-metal catalysts while generating hydrogen as a by-product (Figure 1A). Under the production conditions used in this study, the estimated cost was approximately US$0.07/g, and the field application rate was 0.5 g per Chinese mu. These features suggest that MGN production could be economically compatible with agricultural use, although larger-scale cost analyses will be required to evaluate commercial feasibility.

Protection from UV radiation is another important requirement for agricultural RNA-delivery systems (Lowry et al., 2024). Consistent with the strong UV absorbance of graphene-based materials (Murariu et al., 2021; Kuo et al., 2022), MGNs effectively protected dsRNA from UV-induced photodegradation (Supplemental Figure 2D). MGNs and MSNs also preserved dsRNA integrity more effectively under heat stress than CQDs and PEI under the tested conditions (Supplemental Figure 2E). This difference may reflect physical confinement of dsRNA within the mesoporous structures, although further analyses will be needed to establish the underlying mechanism.

Despite these advantages, MGNs are not inherently biodegradable, raising concerns about their environmental persistence and potential accumulation. Other RNA-delivery nanocarriers may have more favorable degradation profiles. Clay nanosheets and DNA nanostructures are degradable (Mitter et al., 2017b; Zhang et al., 2019), carbon dots can undergo degradation through sunlight exposure and biological activity (Srivastava et al., 2019; Liu et al., 2021), and mesoporous silica nanoparticles degrade through hydrolytic dissolution (Zhang et al., 2024). The long-term fate of MGNs in plants, soils, and associated organisms therefore requires further investigation. Future work should examine their persistence, transport, transformation, and accumulation under realistic agricultural conditions. Surface functionalization may also improve their performance and degradability. Possible approaches include incorporating cleavable or oxidizable groups, introducing defect-rich or hybrid biodegradable structures, and designing stimuli-responsive coatings that promote degradation after cargo release.

The average hydrodynamic diameter of the MGNs was approximately 280 nm, with a range of 140 to 540 nm. This size is larger than that of sub-100-nm particles, which have been associated with pulmonary and capillary accumulation in some biological systems (Chithrani et al., 2006; Nel et al., 2006; Oberdoester, 2010). It also exceeds the commonly reported size-exclusion limit of the plant cell wall, which is approximately 5 to 20 nm (Cunningham et al., 2018; Wang et al., 2019). Intact MGNs are therefore unlikely to enter plant cells readily. Nevertheless, previous studies have shown that relatively large nanocarriers, including 100–200 nm MSNs, and smaller carriers with limited cellular entry, such as 5–20 nm DNA-modified gold nanoparticles, can still mediate nucleic acid delivery in plants (Torney et al., 2007; Zhang et al., 2022). Our results are consistent with the possibility that intact nanoparticle internalization is not essential for MGN-mediated RNA delivery. One potential model is that MGNs remain associated with the leaf surface or apoplast and gradually release dsRNA, which is then taken up by plant cells (Mitter et al., 2017a). Although this sustained-release approach may result in lower cellular uptake, our phenotypic data confirm that it can nevertheless effectively silence S genes. Crucially, the use of larger carriers may reduce the risks of nanotoxicity and bioaccumulation in agricultural ecosystems (Iavicoli et al., 2017), thereby enhancing long-term ecological safety.

Collectively, our findings establish a novel disease resistance strategy based on the silencing of S genes to enhance broad-spectrum disease resistance while avoiding the adverse effects associated with S gene deletion. We synthesized a 3D graphene structure that can efficiently protect and deliver dsRNA and validated the feasibility of this strategy in both model plants and field-grown rice. Given the transient and tunable nature of this gene-silencing approach, it may represent a versatile tool that extends beyond crop protection. This platform could be adapted to modulate other essential traits, such as drought and cold tolerance, offering a promising strategy for precision molecular breeding and sustainable agriculture, although further investigation is warranted.

## Methods

### Preparation of MGNs

MGNs were synthesized in a specialized PECVD chamber. A 3-mm-thick graphite disk housed in a high-purity graphite boat served as the growth substrate. The reaction zone was maintained at 800°C throughout the synthesis. A radiofrequency power of 500 W was applied to generate a stable plasma and enhance the kinetic energy of reactive ions. The chamber pressure was maintained below 0.075 Torr during the 120-min deposition period. Under these plasma-enhanced conditions, hydrocarbon molecules decomposed and nucleated into a 3D carbon framework without the need for external catalysts or templates (Bo et al., 2013). Concurrent etching by the H_2_ plasma contributed to the formation of the disordered mesoporous architecture (Lee et al., 2014). This process has a low environmental footprint and yields high-purity hydrogen gas as a valuable by-product.

To further improve crystallinity and graphitization, the as-synthesized material was subjected to high-temperature post-treatment. Samples were heated to 2,000°C in an induction furnace under high-purity argon with a ramp rate of 10°C/min and maintained at the target temperature for 2 h. After natural cooling to room temperature under an inert atmosphere, the resulting 3D graphene powder was mechanically collected from the substrate for subsequent characterization and dsRNA loading.

### Plant materials and growth conditions

*N*. *benthamiana* and wild-type *Solanum tuberosum* (cv. Desiree) plants were grown in a greenhouse at 25°C and 60% relative humidity under a 16-h light/8-h dark photoperiod for 4 to 6 weeks before experimentation. For virus-induced gene silencing (VIGS) experiments, *N*. *benthamiana* plants were grown at 22°C and 58% relative humidity under the same photoperiod.

Rice (*Oryza sativa* cv. Nipponbare) plants were grown in a growth chamber at 28°C and 80% relative humidity under a 12-h light/12-h dark photoperiod. Field experiments were conducted using plants grown in protected paddy fields.

### RNA extraction and RT–qPCR assays

Total RNA was extracted from *N*. *benthamiana* leaves or *O. sativa* tissues using the RNA-simple Total RNA Kit (Tiangen Biotech) according to the manufacturer’s instructions. First-strand cDNA was synthesized using the HiScript II Q RT SuperMix for qPCR Kit (Vazyme). Quantitative real-time PCR was performed using SYBR Premix Ex Taq (Takara Bio) on an ABI Prism 7500 Fast Real-Time PCR System. Gene expression levels were normalized to *NbEF1α* (a reference gene for *N*. *benthamiana*) and *OsActin* (a reference gene for rice) (Gao et al., 2021; Dong et al., 2025). RT–PCR analyses were performed using three independent biological replicates. Primer sequences are listed in Supplemental Table 1.

### *In vitro* synthesis of dsRNA and preparation of MGN–dsRNA complexes

For dsRNA synthesis, total RNA was extracted from *N*. *benthamiana* or rice and reverse transcribed to cDNA as described above. dsRNA templates were amplified from the resulting cDNA and used for *in vitro* transcription with the T7 RiboMAX Express RNAi System (Promega, USA). The synthesized dsRNA was stored in 1× TBS. Primer sequences are provided in Supplemental Table 1.

MGNs were dispersed in nuclease-free water and sonicated for 30 min to ensure homogeneous dispersion. MGNs and dsRNA were then mixed at a 1:1 mass ratio and incubated at 25°C for 30 min with gentle shaking (40 rpm) to allow dsRNA loading onto the MGNs.

### Characterization of products

The morphology of dsGFP and MGN–dsGFP (final concentration, 100 ng/μl) was examined by TEM using a JEM-2100 microscope (JEOL, Japan). UV-visible absorption spectra of MGNs, dsGFP, and MGN–dsGFP (100 ng/μl) were recorded using a Unico 2800 spectrophotometer (Unico Instruments, USA). Hydrodynamic diameter and zeta potential were measured using a Zetasizer Nano ZS90 system (Malvern Panalytical, UK). Samples contained either MGNs alone (100 ng/μl) or MGN−dsGFP complexes (MGN concentration, 100 ng/μl; mass ratio, 1:1). Measurements were performed in RNase-free 1× TBS (pH 7.5) at 25°C. Electrophoretic mobility was converted to zeta potential using the Smoluchowski model.

### Specific surface area and porosity analysis

To characterize the specific surface area and pore structure, bare MGNs and MGN–dsRNA complexes (final concentration, 100 ng/μl) were lyophilized (freeze-dried) for 48 h to obtain thoroughly dried powder samples. Nitrogen (N_2_) adsorption–desorption isotherms were then measured at 77 K using an automated surface area and porosity analyzer (ASAP 2460; Micromeritics, USA). Before analysis, the lyophilized samples were degassed under a vacuum at 100°C for 6 h. Specific surface area was calculated using the BET method, and pore-size distribution was determined from the desorption branch of the isotherms using the BJH model.

### Gel retardation assay

An agarose gel retardation assay was performed to determine the optimal MGN-to-dsRNA binding ratio. MGNs and dsRNA were mixed at different mass ratios in TBS. To evaluate the effect of ionic strength on complex formation, NaCl was added to achieve final concentrations of 150 or 200 mM. The mixtures were gently vortexed and incubated at room temperature for 30 min to allow MGN–dsRNA complex formation. Samples were then mixed with 10× DNA loading buffer (Vazyme, P022-01) and loaded onto a 1.2% (w/v) agarose gel prepared in 1× Tris-acetate–EDTA (TAE) buffer containing 1×Ultra GelRed (10,000×) nucleic acid stain (Vazyme, GR501-01). Electrophoresis was performed at 100 V for 30 min. Free dsRNA was visualized using a Tanon 5200 gel documentation system. Complete disappearance of the free dsRNA band was considered indicative of complete complex formation between dsRNA and MGN (Li et al., 2022a).

### *In vitro* dsRNA release kinetics assay

The release profile of dsRNA from MGN nanocarriers was evaluated by agarose gel electrophoresis. MGN–dsRNA complexes were prepared at a 1:1 mass ratio, yielding a final dsRNA concentration of 100 ng/μl. Naked dsRNA at the same concentration served as a control. To examine release under different conditions, samples were adjusted to pH 7.5 or pH 5.5 and incubated at 25°C with gentle shaking (40 rpm) (Wang et al., 2023a). At 12, 24, 36, 72, 96, and 120 h, 10-μl aliquots were collected and analyzed by agarose gel electrophoresis. Gel images were quantified using ImageJ by measuring the integrated fluorescence intensity of each dsRNA band. The percentage of released dsRNA was calculated by comparing the band intensity of released dsRNA with that of the corresponding naked dsRNA control at each time point.

### Pathogen inoculation assay

*P*. *capsici* strain LT263 was cultured on 10% V8 vegetable juice agar at 25°C in the dark. *M*. *oryzae* strain Guy11 was cultured on complete agar medium at 25°C. *Xoo* strain PXO99A was grown on peptone sucrose agar at 28°C for 3 days.

For *P*. *capsici* inoculation, 6-week-old *N*. *benthamiana* plants were sprayed with 10 ng/μl MGN–dsRNA, naked dsRNA, MGNs, or H_2_O (control). Twenty-four hours later, 6-mm mycelial plugs taken from the edge of actively growing colonies were placed on the abaxial leaf surface, and 5 μl sterile water was added to maintain humidity.

For *P*. *infestans* inoculation, 6-week-old *S*. *tuberosum* (cv. Desiree) plants were sprayed with double-distilled H_2_O (mock control), bare MGNs, naked ds*StBPA*, or MGN–ds*StBPA* complexes (Li et al., 2025). At 24 h after treatment, plants were sprayed with 10 ml of a *P. infestans* zoospore suspension (4 × 10^4^ spores/ml) (Dong et al., 2025). Plants were then transferred to a climate-controlled chamber and maintained under high humidity to promote infection. Disease symptoms were recorded at 4 dpi, and lesion areas were quantified using ImageJ.

For *Xoo* inoculation, bacterial cells were suspended in sterile water to an optical density of OD_600_ = 0.5 and inoculated onto 2-month-old rice plants at the tillering stage using the leaf-clipping method (Gao et al., 2021). Lesion length was measured at 14 dpi.

For rice blast assays, 10 ng/μl MGN–dsRNA, naked dsRNA, MGNs, or H_2_O (control) was applied to rice plants as described above before inoculation. *M*. *oryzae* Guy11 conidia were suspended at 5 × 10^4^ spores/ml in 0.2% (w/v) gelatin, and 4 ml of the suspension was sprayed onto 2-week-old rice seedlings. Plants were maintained at 25°C and 90% relative humidity in the dark for the first 24 h, followed by a 12-h light/12-h dark photoperiod. Disease severity was evaluated at 7 dpi.

### MST assay

Interactions between MGNs and dsRNA were analyzed by MST on a Monolith NT.Label Free instrument (NanoTemper Technologies, Germany) (Park et al., 2014; Pei et al., 2024). Fluorescently labeled MGNs were prepared at a concentration of 18 nM in nuclease-free water. Serial dilutions of ds*GFP* (0.0317–520 nM) were mixed with the labeled MGNs at a 1:1 volume ratio and incubated for 10 min at room temperature. The reaction mixtures were then loaded into standard NT.Label Free capillaries. MST measurements were performed using 20% LED power and 40% MST power. Dissociation constants (*K*_D_) were determined by curve fitting using the integrated Kd-fit function in NanoTemper Analysis software (version 1.5.41).

### DAB staining for hydrogen peroxide detection

Two-week-old rice seedlings or 4-week-old *N*. *benthamiana* plants were sprayed with the indicated compounds at a final concentration of 10 ng/μl. At 1, 3, 5, or 7 days after treatment, leaves were excised and immersed in 1 mg/ml DAB solution (Sigma-Aldrich) at 25°C for 8 h to detect hydrogen peroxide accumulation. The stained leaves were then cleared by boiling in absolute ethanol until the chlorophyll was completely removed. Cleared leaves were mounted in 30% glycerol for imaging (Ai et al., 2023).

### *Agrobacterium*-mediated transient expression in *N*. *benthamiana*

For transient expression assays, leaves of 6-week-old *N*. *benthamiana* plants were infiltrated with *Agrobacterium tumefaciens* strain GV3101 carrying the indicated plant expression constructs. GV3101 cells transformed by electroporation were cultured overnight at 28°C in Luria–Bertani medium supplemented with 50 μg/ml kanamycin and 25 μg/ml rifampicin. Bacterial cells were collected by centrifugation at 4,000× *g* for 4 min at room temperature and washed three times with sterile infiltration buffer (10 mM MgCl_2_, 10 mM MES [pH 5.7], and 200 μM acetosyringone). The final bacterial suspension was adjusted to an OD_600_ of 0.4–0.6 in fresh infiltration buffer. Leaf samples were collected at 48 h post-infiltration for subsequent analyses.

### Protoplast extraction isolation

Leaf strips from *N*. *benthamiana* were incubated in an enzyme solution containing 0.4 M mannitol, 20 mM MES (pH 5.7), 10 mM CaCl_2_, 20 mM KCl, 10 mg/ml β-1,3-glucanase, and 5 mg/ml cellulase at 25°C with gentle shaking (100 rpm) for 40 min to release protoplasts.

### Confocal microscopy

For dsRNA-delivery assays, dsRNA was fluorescently labeled with fluorescein-12-UTP (Beyotime, cat. no. D7338) according to the manufacturer’s instructions. Leaf disks were collected from *N*. *benthamiana* or rice plants 12 h after spraying with fluorescently labeled MGN−dsRNA complexes or naked dsRNA (final concentration of 200 ng/μl). The disks were mounted in distilled water and imaged using a Leica TCS SP8 confocal laser-scanning microscope equipped with a 20× water-immersion objective. Fluorescein was excited at 488 nm, and green fluorescence was detected within the corresponding emission range. For dsRNA-delivery assays in protoplasts, *N. benthamiana* protoplasts were incubated for 8 h under low-light conditions with fluorescein-12-UTP-labeled dsGFP or MGN–dsGFP complexes (final concentration of 200 ng/μl). Cellular fluorescence was then imaged using a Leica TCS SP8 confocal laser-scanning microscope.

To examine the localization of dsRNA relative to the plasma membrane, AtFLS2-mCherry was transiently expressed in *N. benthamiana* leaves by *Agrobacterium-mediated infiltration* (Zhao et al., 2024). At 36 hpi, leaves were sprayed with 200 ng/μl fluorescently labeled dsGFP or MGN–dsGFP. Fluorescence was imaged 12 h after treatment using a Leica TCS SP8 confocal laser-scanning microscope. Fluorescein-12-UTP and mCherry were excited at 488 and 561 nm, respectively. Emission was detected at 500 to 530 nm for fluorescein and 590 to 620 nm for mCherry. Images were acquired using sequential scanning to minimize signal bleed-through. Colocalization between AtFLS2-mCherry and dsGFP signals was analyzed using ImageJ.

To assess dsRNA localization in mesophyll cells, chloroplast autofluorescence was used as an endogenous marker. All other procedures were performed as described above.

### Stability test of MGN–dsRNA complexes

To evaluate the stability of naked dsRNA and MGN–dsRNA complexes under different stress conditions, samples were prepared at a final dsRNA concentration of 100 ng/μl and subjected to four treatments: thermal stress at 95°C; continuous UV irradiation at 253.7 nm; enzymatic degradation with 1 U/μl dsRNase (MCE, HY-KE7056-100 U) in 10× RNase III reaction buffer at 37°C for 30 min; or exposure to outdoor conditions, including natural sunlight and an ambient temperature of 25°C ± 2°C (Jiang et al., 2026).

Before gel electrophoresis, MGN–dsRNA samples were supplemented with Triton X-100 to a final concentration of 1% and vortexed vigorously for 10 min to release the bound dsRNA. Samples were then separated on a 1% agarose gel (prepared in 1× TAE buffer) at 100 V for 30 min. Gels were stained with ethidium bromide or SYBR Safe and imaged using a Tanon 5200 imaging system. Band intensities were quantified using ImageJ and normalized to those of untreated controls (*n* = 3).

### VIGS assays in *N*. *benthamiana*

For VIGS experiments, the tobacco rattle virus vectors pTRV1 and pTRV2 (including pTRV2-*GUS* and pTRV2-*BIR1*) were introduced into *A. tumefaciens* strain GV3101 by electroporation. Equal volumes of *Agrobacterium* suspensions carrying pTRV1 and the corresponding pTRV2 construct, each adjusted to an OD_600_ = 0.5, were mixed and infiltrated into the true leaves of 2-week-old *N*. *benthamiana* plants. Plants were maintained at 22°C under a 16-h light/8-h dark photoperiod for 4 weeks.

### Field trials and efficacy evaluation

Field trials were conducted at two sites in China to evaluate the efficacy of MGN−dsROD1 under agricultural conditions: Wangzhong Village, Long’an County, Nanning, Guangxi Province (23.0858°N, 107.8245°E), where the soil was classified as ferric Acrisol; and Longlou Village, Sanya, Hainan Province (18.4014°N, 109.7507°E), where the soil was classified as gleyic Regosols. *O. sativa* cv. Nipponbare was used in both trials. Each treatment comprised four plots of 30 m^2^. Four foliar applications were performed during key developmental stages. The first application was conducted at the late tillering stage, followed by a second application 10 days later. The third application was performed at the early heading stage, and the fourth was applied 10 days thereafter. The dsRNA concentration was maintained at 10 ng/μl for all treatments. At the end of the trial, disease incidence and key agronomic traits were recorded to evaluate the protective efficacy of MGN–dsROD1 and its effects on rice growth and yield components.

### Assessment of the effects of MGNs on rice seedling growth and physiological traits

The effects of MGNs on rice seedling growth were assessed using a hydroponic assay with *O. sativa* (cv. Nipponbare). Surface-sterilized seeds were germinated for 3 days and then precultured in standard nutrient solution for 7 days. On day 10, the medium was replaced with fresh nutrient solution containing MGNs (prepared from a 1 mg/ml stock solution) at final concentrations of 0, 2, 5, 10, 20, or 40 ng/μl. To minimize nanoparticle sedimentation, the solutions were gently agitated once daily. Each treatment included three biological replicates, with 10 seedlings per replicate.

After 7 days of continuous MGN exposure (corresponding to day 17), plant height and primary root length were measured. Plant height was measured from the stem base to the tip of the longest leaf. Relative chlorophyll content was determined non-destructively in the second fully expanded leaf from the apex using a SPAD-502 meter.

### Environmental biosafety and toxicity assessments

To evaluate the environmental biocompatibility and non-target toxicity of MGNs, toxicity assays were performed using representative organisms from terrestrial and aquatic ecosystems.

For the terrestrial toxicity assay, adult earthworms were maintained in soil irrigated with MGN solutions at final concentrations of 0, 2, 5, 10, 20, or 40 ng/μl (Li et al., 2021a). The commercial insecticide Phoxim was applied at 10 ng/μl as a positive control. Earthworm survival was recorded at 7 and 14 days after treatment. Each treatment included 20 earthworms, and the experiment was performed with three independent biological replicates.

For the aquatic acute toxicity assay, zebrafish (*Danio rerio*) were continuously exposed to water containing MGNs at the same concentration range. Survival was recorded at 24, 48, and 72 h after exposure. Each treatment included 15 zebrafish, and the experiment was independently repeated three times under standard laboratory conditions (Niu et al., 2024).

### Comparative phytotoxicity and transcriptional response analysis

To compare the phytotoxicity of MGNs with that of other nanocarriers and assess their effects on stress-responsive gene expression, an *in planta* assay was performed using *O. sativa* (cv. Nipponbare). Leaves were sprayed with MGNs, CQDs, PEI, or MSNs at a final concentration of 10 ng/μl. Sterile water was used as a mock control.

At 3 days after treatment, the net photosynthetic rate of the treated leaves was measured using a portable photosynthesis system (LI-COR LI-6400XT). Measurements were collected from more than 30 individual leaves per treatment, with three technical measurements taken per leaf. Leaf tissues from the corresponding treatments were collected at the same time, immediately frozen in liquid nitrogen, and used for RNA extraction. Genes for expression analysis were identified by searching the Nipponbare genome using the *Arabidopsis thaliana* stress-responsive genes (*AtGLR29*, *AtPR1*, and *AtWRKY40*) as queries (Bjornson et al., 2021). *OsGLR28*, *OsPR1α*, and *OsWRKY71* were selected as the corresponding rice genes and used for expression analysis (Shang et al., 2023). RT-qPCR analyses were performed using three independent biological replicates.

### Phyllosphere microbiome profiling

To assess the potential effects of MGNs on plant-associated microbial communities, phyllosphere microbiome profiling was performed using treated rice leaves. Rice plants were sprayed with either 10 ng/μl MGNs or sterile water (as a mock control). At 10 days after treatment, leaves were carefully collected using sterile procedures to minimize external contamination. Samples were immediately frozen in liquid nitrogen and sent to Sinong (Nanjing, China) for microbial genomic DNA extraction, amplicon library preparation, and high-throughput sequencing.

### Statistical analysis

Data are presented as the means ± standard deviation (SD) from at least three biological replicates unless otherwise indicated. Comparisons between two groups were performed using Student’s *t*-test. Comparisons among multiple groups were performed using one-way analysis of variance (ANOVA) followed by Tukey’s multiple-comparison test. Statistical significance is indicated by asterisks (∗*p* < 0.05 and ∗∗*p <* 0.01) or by different lowercase letters, as specified in the figure legends. Statistical methods, sample sizes, and definitions of replicates are provided in the corresponding figure legends.

## Data and code availability

All data supporting the findings of this study are available within the article and its Supplemental information.

## Funding

This work was supported by the 10.13039/501100001809National Natural Science Foundation of China (32402315 and 32230089), the 10.13039/501100012226Fundamental Research Funds for the Central Universities (QTPY2026011), the 10.13039/501100004608Natural Science Foundation of Jiangsu Province, China (BK20230984), and the 10.13039/501100012453China Agricultural Research System (CARS-21).

## Acknowledgments

The authors declare no conflict of interest.

## Author contributions

D.D., G.A., J.C., and W.L. conceived and designed the project, jointly performed data analysis, and wrote the manuscript; W.L., Y.Y., C.W., Y.H., Z.C., Z.L., J.M., Y.R., X.L., D.L., and Z.Z. performed the experiments; G.A., W.L., Y.Y., and C.W. analyzed data; and D.D., G.A., W.L., and C.W. wrote and modified the manuscript. All authors read and approved the final manuscript.

## Supplemental information

Document S1. Supplemental Figures 1–7 and Supplemental Table 1

Document S2. Article plus supplemental information
