# Supplementary material for: 3D mesoporous graphene nanoparticle-mediated transient silencing of susceptibility genes confers broad-spectrum disease resistance in crops
Source: Plant Commun. 2026 Jun 29;7(9):101983. doi: 10.1016/j.xplc.2026.101983 (PMC13589569; doi:10.1016/j.xplc.2026.101983)
Supplement: Supplementary file 1 [file mmc1.pdf]

Plant Communications, Volume

Supplemental information

3D mesoporous graphene nanoparticle-mediated transient silencing of susceptibility genes confers broad-spectrum disease resistance in crops

Wangshan Lu, Yule Ye, Chao Wang, Yizhou Hao, Zhaojie Chen, Zhuoyi Lin, Jinbiao Ma, Yueyi Ren, Xinyu Liu, Dongong Niu, Zhengguang Zhang, Jianhao Chen, Gan Ai, and Daolong Dou

Supplementary Information

Three-dimensional mesoporous graphene nanoparticle-mediated transient silencing of susceptibility genes confers broad-spectrum disease resistance in crops

Wangshan Lu[1#], Yule Ye[1#], Chao Wang[1], Yizhou Hao[2], Zhaojie Chen[3], Zhuoyi Lin[3], Jinbiao Ma[4], Yueyi Ren[1], Xinyu Liu[1], Dongong Niu[1], Zhengguang Zhang[1], Jianhao Chen[5*], Gan Ai[1*], Daolong Dou[1*]

[1]State Key Laboratory of Agricultural and Forestry Biosecurity, College of Plant Protection, Academy for Advanced Interdisciplinary Studies, Nanjing Agricultural University, Nanjing, China

[2]Guangzhou Moxi Technology Co Ltd. Guangzhou 510535, China

[3]Guangxi Key Laboratory of Agric-Environment and Agric-Products Safety, College of Agriculture, Guangxi University, Nanning, Guangxi, 530004, China

[4]State Key Laboratory of Wheat Improvement, College of Life Sciences, Shandong Agricultural University, Tai'an, 271018, China

[5]International Center for Quantum Materials, School of Physics, Peking University, Beijing 100871, China

#These authors contributed equally.

*Corresponding author: Daolong dou: ddou@njau.edu.cn, Gan Ai: ganai@njau.edu.cn and Jianhao Chen: chenjianhao@pku.edu.cn.

Supplemental Table 1. Primers used in this study

Name	Sequence (5' to 3')	Usage
qRT-PCRGFP-F	CACATGAAGCAGCACGACTT	qRT-PCR
qRT-PCRGFP-R	AGTTCACCTTGATGCCGTTC	
qRT-PCRNbActin-F	CTGGGTTTGCTGGAGATGAT	
qRT-PCRNbActin-R	CATCTCCCACGTAGGCATCT	
qRTPCR-BIR1-F	GGACATGCTAAATGGCACCT	
qRTPCR-BIR1-R	ATGAGCCTAGCGAGTCCAAA	
qRTPCR-ROD1-F	GTTCATGTCCAAGAACGGCG	
qRTPCR-ROD1-R	GCCTCGGGATCAGCGAATAA	
qRTPCR-OsActin-F	TGTATGCCAGTGGTCGTACCA	
qRTPCR-OsActin-R	CCAGCAAGGTCGAGACGAA	
qRTPCR-OsPR1a-F	CGTCTTCATCACCTGCAACTACTC	
qRTPCR-OsPR1a-R	CATGCATAAACACGTAGCATAGC	
qRTPCR-OsWRKY71-F	CACCAGCAGCCTCTCAAGAA	
qRTPCR-OsWRKY71-R	GAAGTAGGCTCTTGGGCAGG	
qRTPCR-OsGLR28-F	GCAAAGGGTGGTATTGCTGC	
qRTPCR-OsGLR28-R	ATACTTGGACTTGGCAGCCC	
qRTPCR-StBPA-F	ACTACCTCAGCCACAGTTGC	
qRTPCR-StBPA-R	GGTTTGGGACTCGGGATGTT	
qRTPCR-StActin-F	AGCACCCTGTTCTGCTCACT	
qRTPCR-StActin-R	GCACAGCCTGAATAGCAACA	
dsGFP-F	TAATACGACTCACTATAGGGAGGGCGAGGAGCTGTTCACC	dsRNA synthesis
dsGFP-R	TAATACGACTCACTATAGGGCGTCCTTGAAGAAGATGGTG	
dsNbBIR1-F	TAATACGACTCACTATAGGGACCTCTGTCACCAAAGCTCC	
dsNbBIR1-R	TAATACGACTCACTATAGGGTTGATGTCCGCCATCATCAC	
dsROD1-F	TAATACGACTCACTATAGGGAGGAGGAGGGGTGGCGCTGG	
dsROD1-R	TAATACGACTCACTATAGGGCCACGGGGACGACGAGCGTG	
dsStBPA-F	TAATACGACTCACTATAGGGGGATCTGCTATTCAAAAGGC	
dsStBPA-R	TAATACGACTCACTATAGGGGGCGGATCCAGCATTGCTAA	

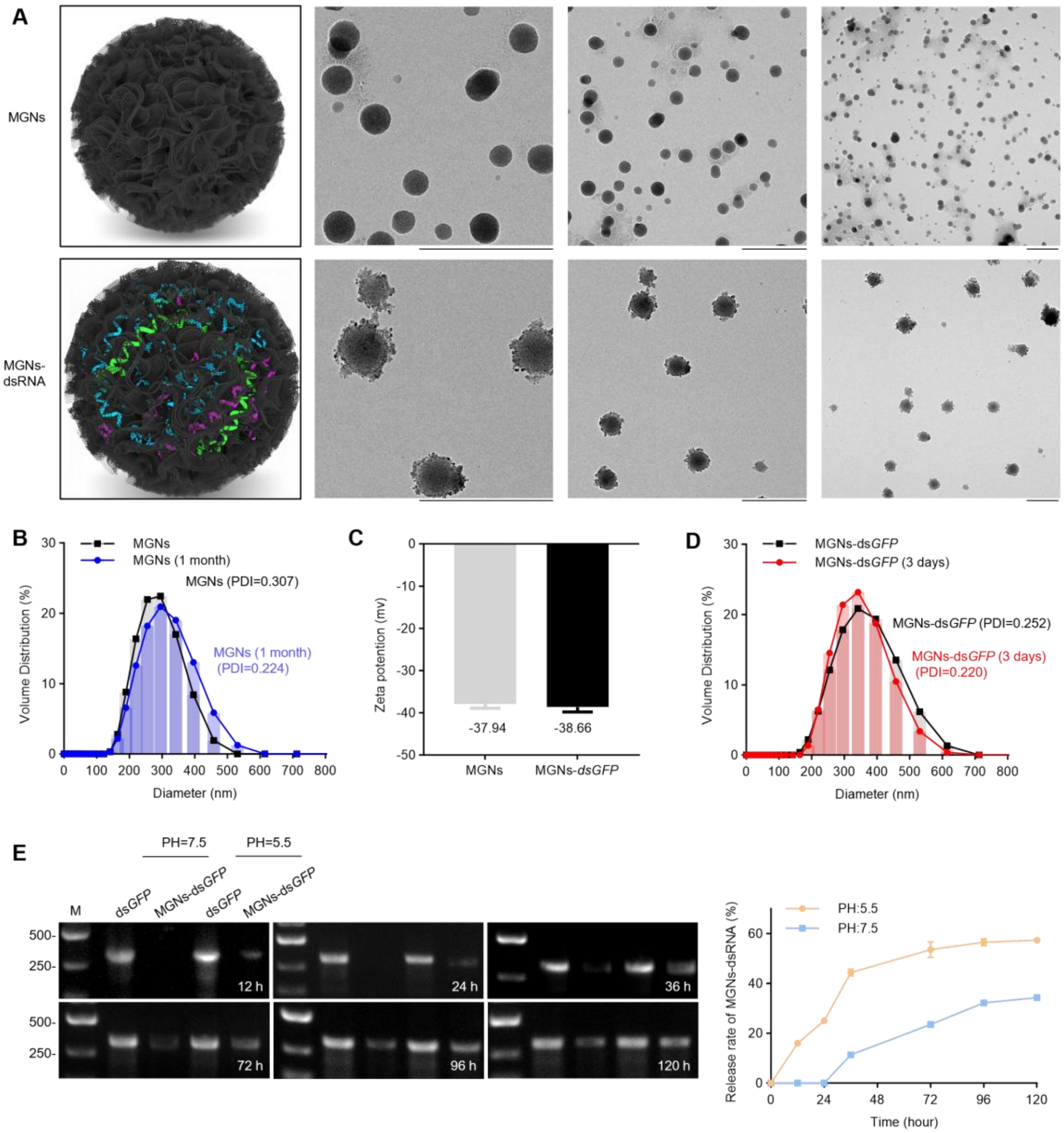

Supplemental Figure 1. Loading of dsRNA onto MGNs.

(A) TEM images showing the morphologies of MGNs and MGNs-dsRNA complexes Computer simulations of MGNs and MGNs-dsRNA complexes are shown at left while TEM images are shown in right. Bar = 1 μm. (B) Comparison of the hydrodynamic diameters between freshly synthesized MGNs and MGNs stored at 4°C for one month. (C) Zeta potential analysis of MGNs and MGNs-dsRNA complexes. (D) Comparison of the hydrodynamic diameters between freshly prepared MGNs-dsRNA complexes and those stored at 4°C for 3 days. The MGNs-dsRNA complexes remained highly stable after 3 days of storage at 4°C. (E) Agarose gel electrophoresis assay evaluating the dsRNA release kinetics from MGNs-ds*GFP* complexes in aqueous solutions at pH 7.5 and pH 5.5. Naked ds*GFP* (50 ng/μL) was used as a control. The line graph on the right presents the quantitative analysis of the gel electrophoresis shown on the left. Each data point represents the fluorescence intensity of the released ds*GFP* band expressed as a percentage of the control ds*GFP* band, with three independent replicates for each time point.

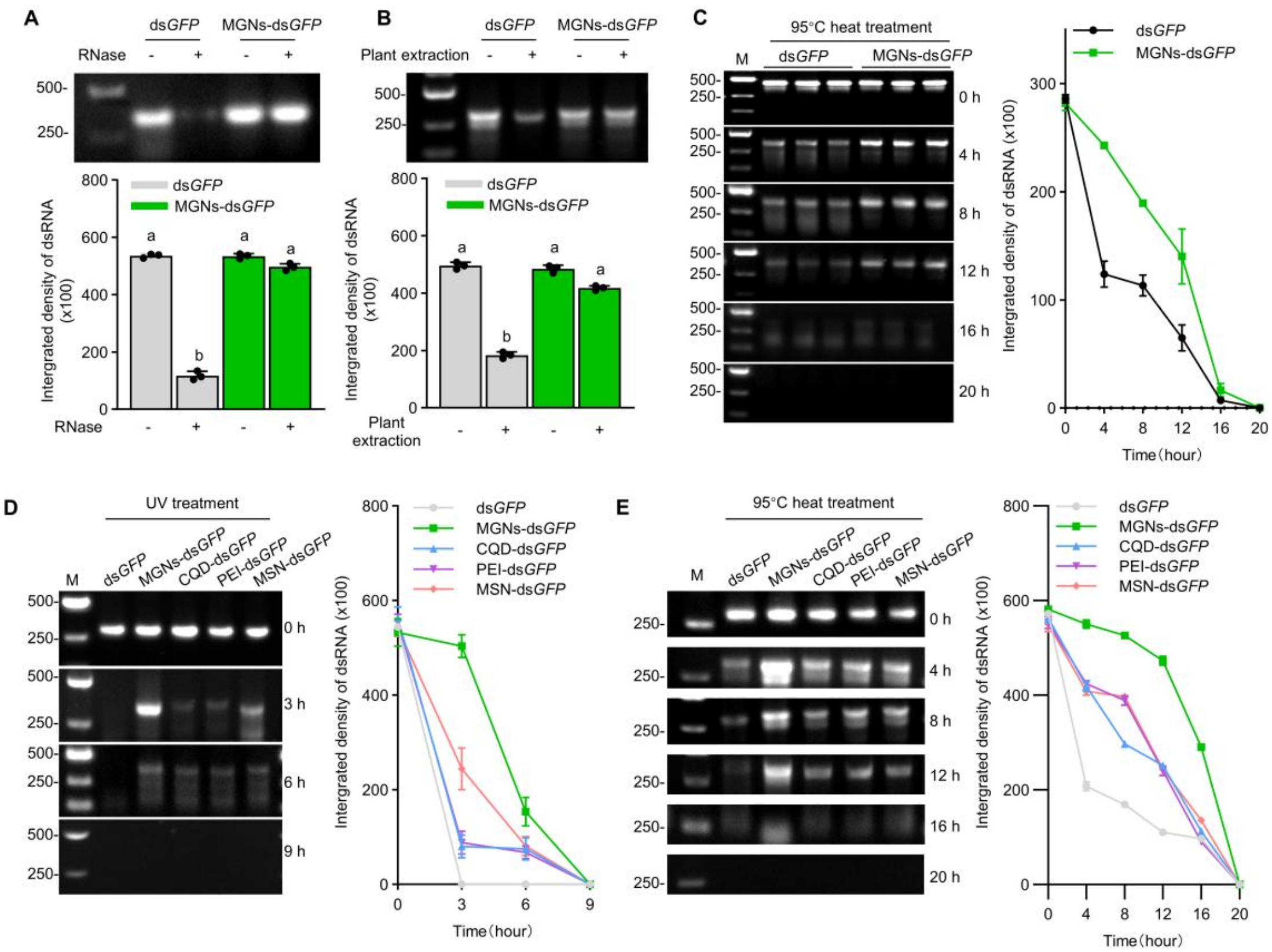

Supplemental Figure 2. MGNs-mediated protection of dsRNA.
(A,B) RNase and plant extraction protection assay. Naked ds*GFP* and MGNs ds*GFP* complexes (final ds*GFP* concentration: 100 ng/μL) were treated with 1 U/μL dsRNase (A) for 30 min or incubated in total protein extracts from *Nicotiana benthamiana* leaves for 12 h (B), and the integrity of the nucleic acids was subsequently evaluated by agarose gel electrophoresis. The corresponding bar chart displays the quantitative fluorescence intensity of the nucleic acid bands. Data are means ± SD (n = 3, one-way ANOVA, P < 0.05). (C) Thermal stability of naked ds*GFP* and MGNs-ds*GFP* complexes. Samples (final ds*GFP* concentration: 100 ng/μL) were subjected to denaturation at 95°C in a thermal cycler for 0, 4, 8, 12, 16, and 20 h. The integrity of the complexes at each time point was evaluated by agarose gel electrophoresis. The line graph on the right illustrates the corresponding fluorescence intensity of the nucleic acid bands on the left. Data are means ± SD (n = 3). (D) UV tolerance assay. Naked ds*GFP* and various nanomaterial-ds*GFP* complexes (MGNs-ds*GFP*, CQD-ds*GFP*, PEI-ds*GFP*, and MSN-ds*GFP*) were subjected to direct UV irradiation in an enclosed chamber for 0, 3, 6, and 9 h, followed by agarose gel electrophoresis. Data are means ± SD (n = 3). (E) Comparative thermal stability of various nanomaterial carriers. Naked ds*GFP*, MGNs-ds*GFP*, CQD-ds*GFP*, PEI-ds*GFP*, and MSN-ds*GFP* complexes were denatured at 95°C for 0, 4, 8, 12, 16, and 20 h, followed by agarose gel electrophoresis at each designated time point. The line graph on the right displays the quantitative fluorescence intensity of the respective nucleic acid bands on the left. Data are means ± SD (n = 3).

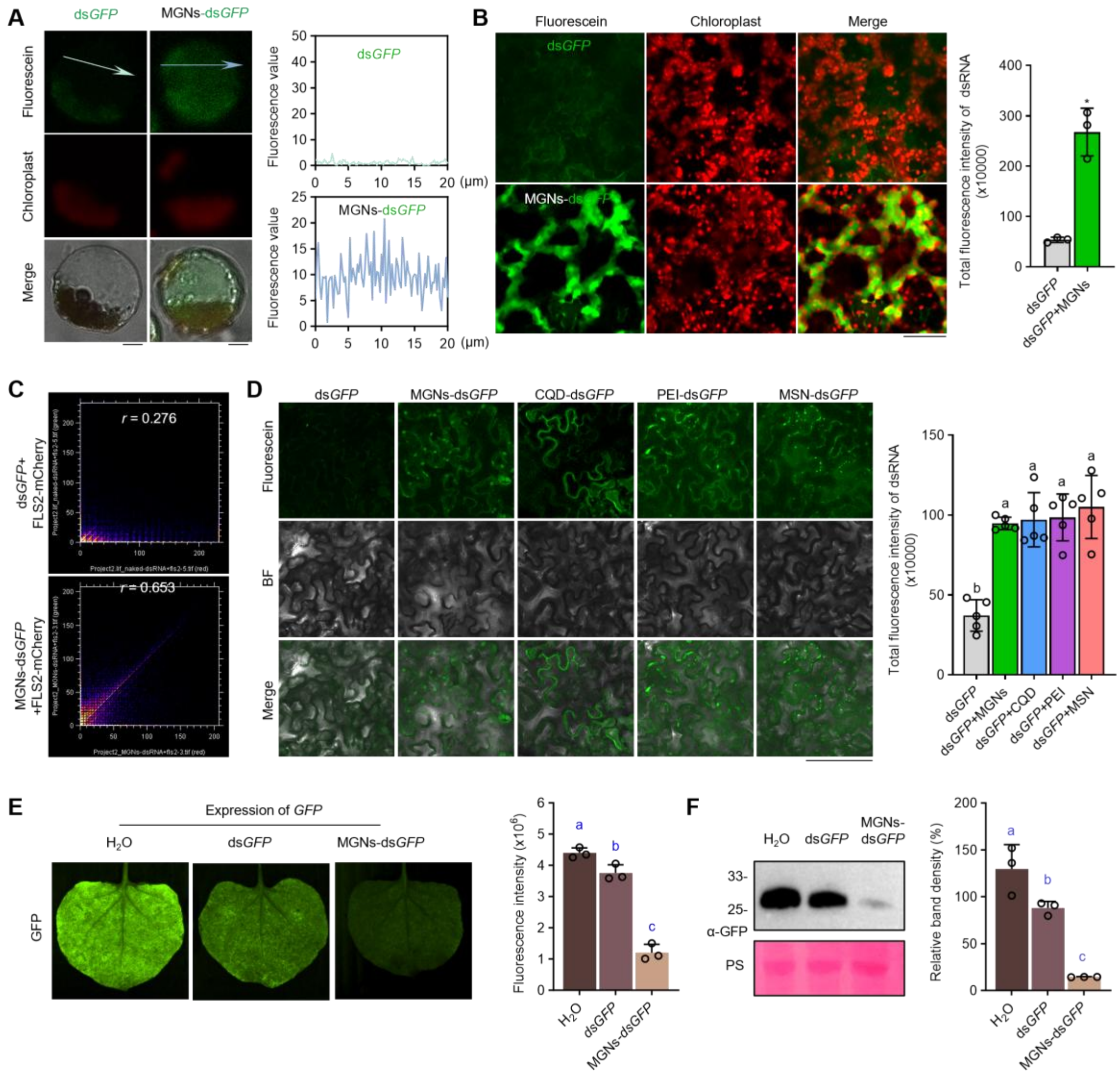

Supplemental Figure 3. Graphene-mediated delivery of dsRNA.

(A) Cellular uptake assay in protoplasts. Protoplasts isolated from *N. benthamiana* were co-incubated with fluorescently labeled naked ds*GFP* or MGNs-ds*GFP* complexes for 8 h and the fluorescence signals was observed under a confocal microscope. The line profile graph illustrates the fluorescence intensity across the trans-sectional line indicated by the arrow in the left panels. Bar = 10 µm. (B) *In planta* internalization of dsRNA. *N. benthamiana* leaves were foliar-sprayed with fluorescently labeled naked ds*GFP* or MGNs-ds*GFP* complexes (ds*GFP* concentration: 200 ng/µL). Chloroplast autofluorescence was used as a marker to delineate mesophyll cells. The bar chart presents the statistical analysis of total green fluorescence intensity from the images. Data are means ± SD (n = 3, student's t-test, *P < 0.05). Bar = 75 µm. (C) Quantitative co-localization analysis of the fluorescence signals from the confocal images in Figure 2C. Analysis was performed using ImageJ software. (D) Comparative delivery efficiency of different nanomaterials. *N. benthamiana* leaves were sprayed with naked ds*GFP*, MGNs-ds*GFP*, CQD-ds*GFP*, PEI-ds*GFP*, and MSN-ds*GFP* complexes. Data are means ± SD (n = 5, one-way ANOVA, P < 0.05). Bar = 100 µm. (E) Silencing of *GFP* by MGNs-ds*GFP*. Data are means ± SD (n = 3, one-way ANOVA, P < 0.05). (F) Western blot analysis. Total proteins were extracted from the *N. benthamiana* leaves shown in Figure 2D, and Western blotting was performed using an anti-GFP primary antibody to evaluate the in planta GFP protein expression levels. The bar chart on the right represents the densitometric quantification of the corresponding protein bands. Data are means ± SD (n = 3, one-way ANOVA, P < 0.05).

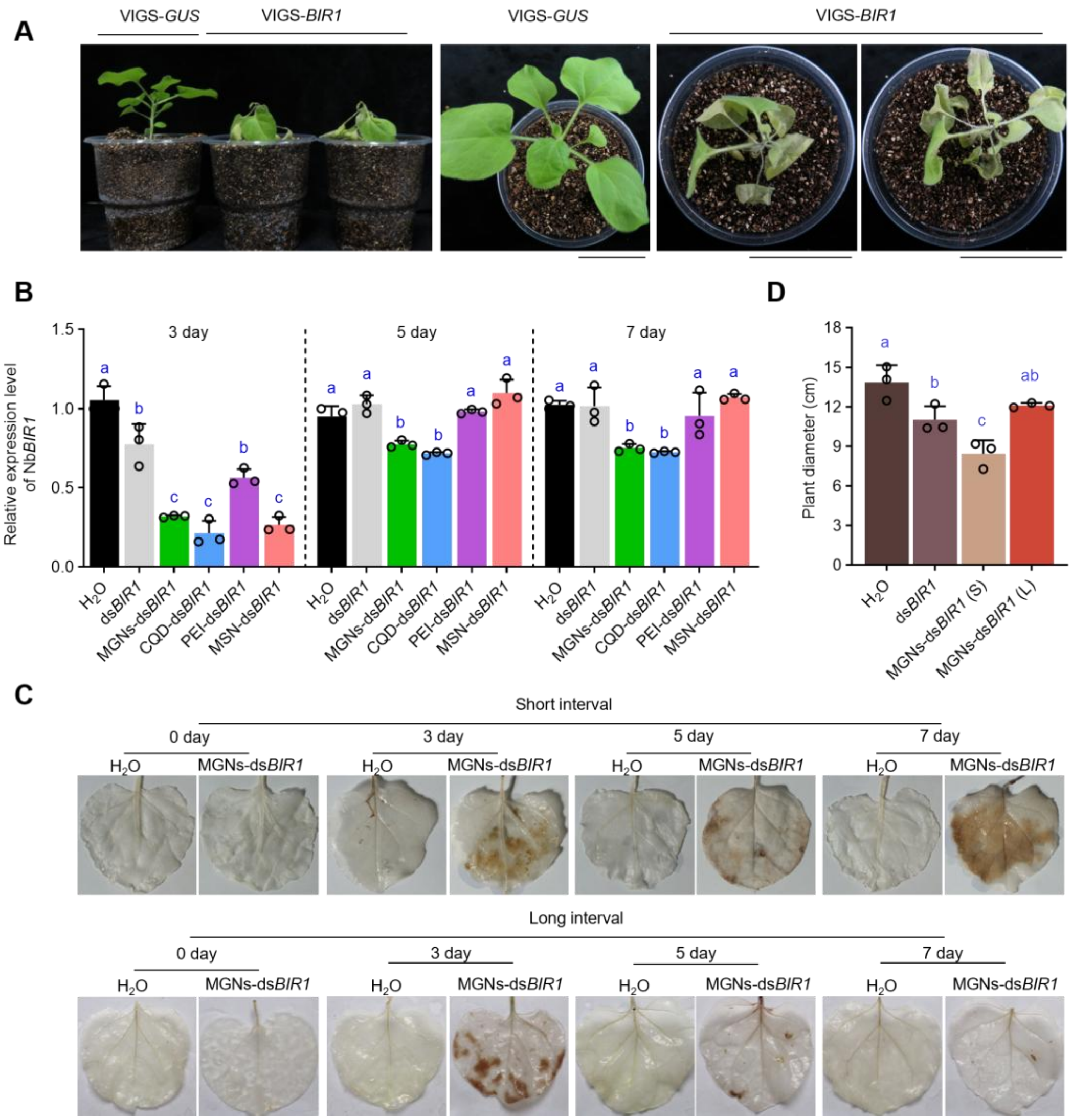

Supplemental Figure 4. Silencing efficiency of MGNs-dsRNA complexes in *N. benthamiana*.

(A) Macroscopic phenotypes of *N. benthamiana* plants following silencing of *NbBIR1*. *BIR1*-silenced plants exhibited a pronounced autoimmune phenotype. Bar = 4 cm. (B) qRT-PCR analysis of *NbBIR1*. Data are means ± SD (n = 3, one-way ANOVA, $P < 0.05$). (C) Visualization of ROS accumulation via DAB staining. *N. benthamiana* leaves were sprayed with H_2O or MGNs-ds*BIR1* complexes (ds*BIR1* concentration: 10 ng/μL) at short (every 3 days) or long (every 7 days) intervals. Leaf samples were collected, stained with DAB, and decolorized for photography at 1, 3, 5, and 7 days after the initial application. (D) Statistical differences of plant diameter from Figure 2F. Data are means ± SD (n = 3, one-way ANOVA, $P < 0.05$).

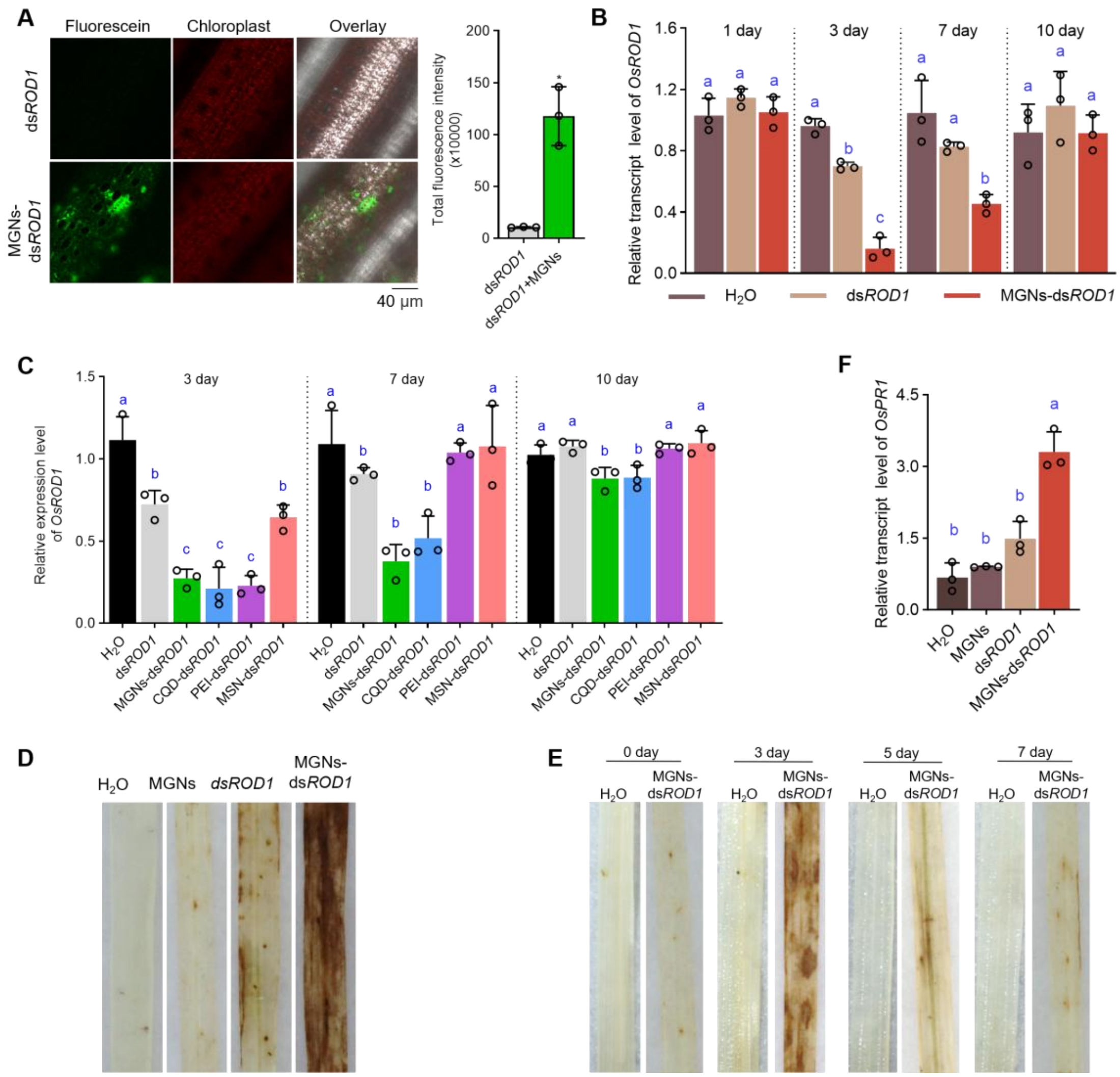

Supplemental Figure 5. Silencing efficiency of MGNs-dsRNA complexes in rice.
(A) dsRNA delivery efficiency of MGNs on rice leaves. Total fluorescence values is shown in right (mean ± SD, n = 3 biological replicates, student's t-test, *P < 0.05). MGNs significantly enhanced the delivery efficiency of dsRNA in rice leaves. Bar = 40 µm. (B) qRT-PCR analysis of *ROD1* transcript levels. The *ROD1* transcript levels of rice leaves at 1, 3, 5, and 7 days post-spraying with H_2O, naked ds*ROD1*, or MGNs-ds*ROD1* complexes were analyzed. Data are means ± SD (n = 3, one-way ANOVA, P < 0.05). (C) qRT-PCR analysis of *ROD1* transcript levels in rice leaves post-spraying with H_2O, naked ds*ROD1*, MGNs-ds*ROD1*, CQD-ds*ROD1*, PEI-ds*ROD1*, and MSN-ds*ROD1* complexes. Data are means ± SD (n = 3, one-way ANOVA, P < 0.05). (D) DAB staining assay. Rice leaves were sprayed with H_2O, MGNs, naked-ds*ROD1* or MGNs-ds*ROD1* complexes (ds*ROD1* concentration: 10 ng/µL). Leaf samples were collected, stained with DAB, and decolorized for photography at 3 days. (E) Time course DAB staining. Rice leaves were sprayed with water or MGNs-ds*ROD1* complexes (ds*ROD1* concentration: 10 ng/µL). Leaf samples were collected, stained with DAB, and decolorized for photography at 1, 3, 5, and 7 days. (F) Transcript levels of *PR1a* in indicated rice leaves. Data are means ± SD (n = 3 biological replicates; one-way ANOVA, P < 0.05).

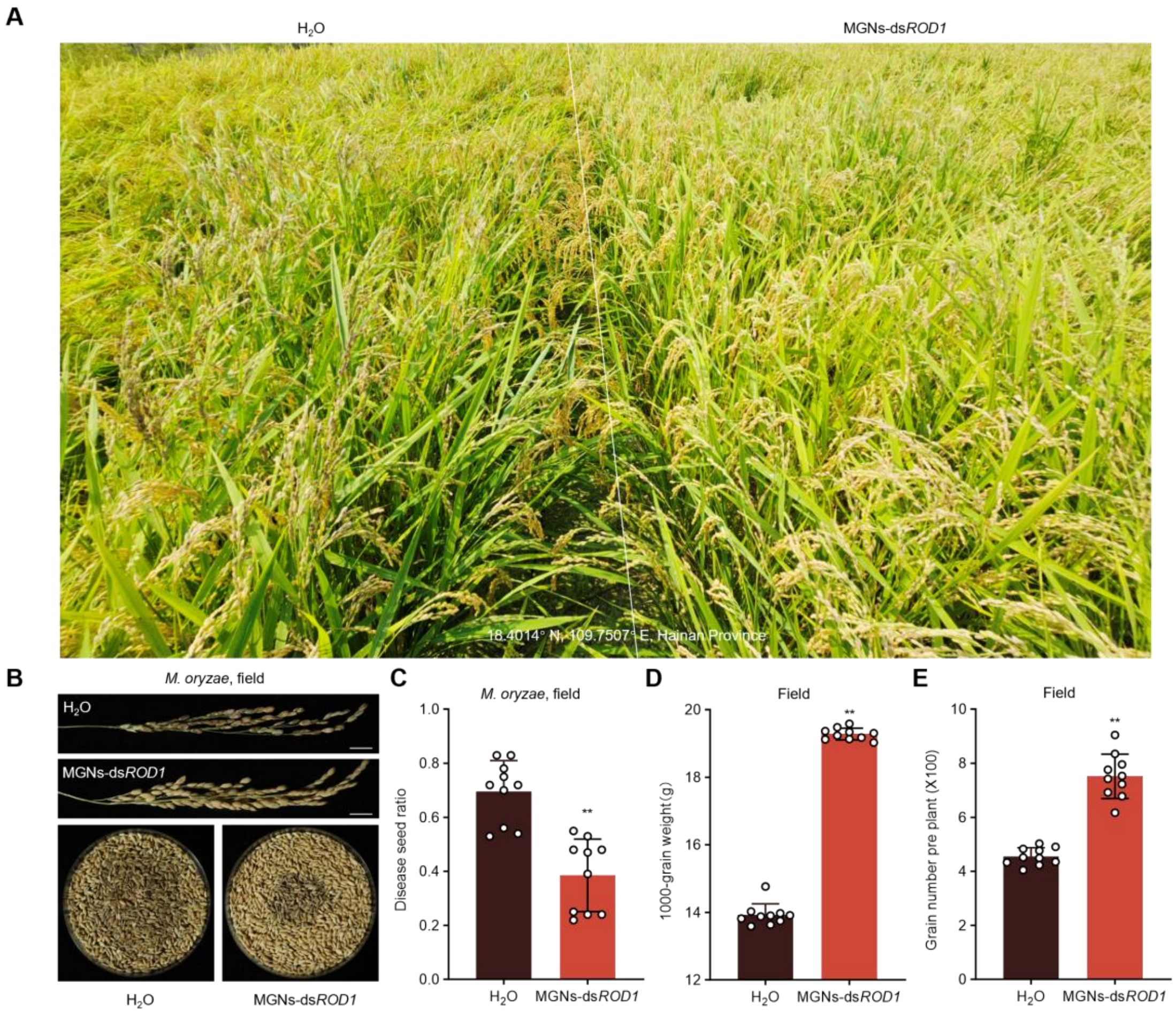

Supplemental Figure 6. MGNs-ds*ROD1* promotes rice resistance in field.
(A) Field blast resistance in rice plants sprayed with H_2O or 10 ng/μL MGNs-ds*ROD1* in the natural nursery. Representative photo is shown. (B,C) Disease phenotypes of rice panicles infected by rice blast in the field. Disease seed ratio caused by *M. oryzae* are shown in (C) (mean ± SD, n = 30 biological replicates, Student's t-test, $^{**}P < 0.01$). Bar = 1 cm. (D,E) Rice yield from (A) increased by MGNs-ds*ROD1*. 1000-grain weight (D) and Number of grains per plant (E) are shown (mean ± SD, $P < 0.05$, one-way ANOVA).

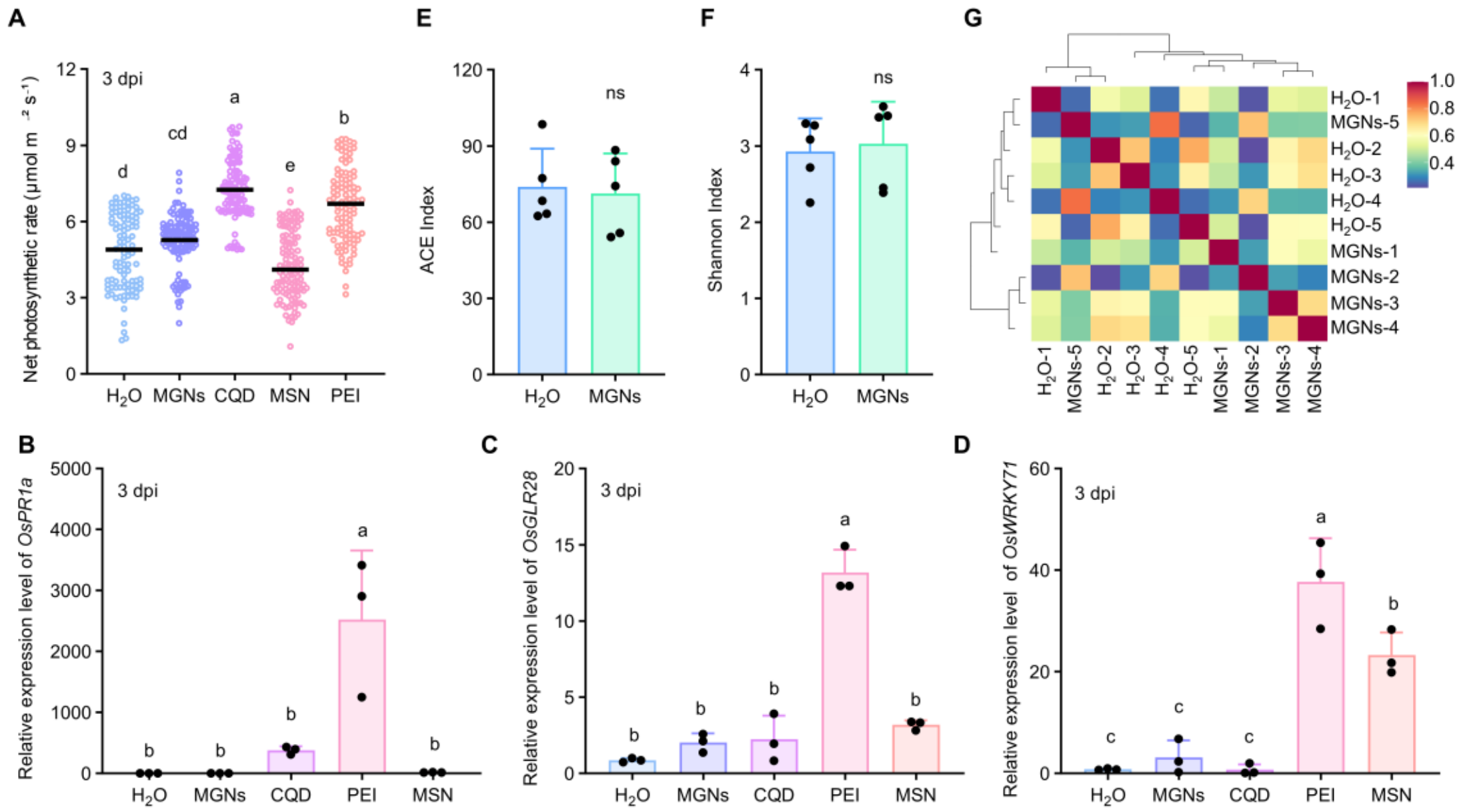

Supplemental Figure 7. Biosafety evaluation of graphene.

(A) Effects of different nanocarriers on the net photosynthetic rate of rice leaves. Leaves were foliar-sprayed with H_2O, or 10 ng/μL of MGNs, CQD, MSN, and PEI. The net photosynthetic rate was measured at 3 dpi. The horizontal lines represent the mean values of each group. Different lowercase letters indicate statistically significant differences ($n > 99$, one-way ANOVA, $P < 0.05$). **(B-D) Relative expression levels of stress-related genes in rice leaves treated with different nanomaterials.** Leaves were foliar-sprayed with H_2O or the indicated nanomaterials (MGNs, CQD, PEI, and MSN). The transcript levels of *OsPR1* (B), *OsGLR28a* (C), and *OsWRKY71* (D) were quantified by RT-qPCR at 3 dpi. Data are presented as means ± SD ($n = 3$ independent biological replicates, one-way ANOVA, $P < 0.05$). **(E,F) Alpha diversity of the bacterial community in the rice phyllosphere.** The ACE index (E) and Shannon index (F) of leaf-associated bacteria was evaluated after foliar spraying with H_2O or 10 ng/μL MGNs. Data are presented as means ± SD ($n = 5$, ns, not significant, student's t-test). **(G) Hierarchical clustering heatmap evaluating sample similarity.** Pairwise correlation analysis of the leaf-associated bacterial communities between the H_2O and MGNs treatments ($n = 5$). The color gradient from blue to red reflects the degree of correlation from low to high (0.2–1.0).
